# Type I IFN signaling limits hemorrhage-like disease after infection with Japanese encephalitis virus through modulating a prerequisite infection of CD11b^+^Ly-6C^+^ monocytes

**DOI:** 10.1186/s12974-021-02180-5

**Published:** 2021-06-15

**Authors:** Ajit Mahadev Patil, Jin Young Choi, Seong Ok Park, Erdenebelig Uyangaa, Bumseok Kim, Koanhoi Kim, Seong Kug Eo

**Affiliations:** 1 College of Veterinary Medicine and Bio-Safety Research Institute, Jeonbuk National University, Iksan, 54596 Republic of Korea; 2 Department of Pharmacology, School of Medicine, Pusan National University, Yangsan, 50612 Republic of Korea

**Keywords:** Type I IFN, Japanese encephalitis, Monocytes, Hemorrhagic disease, Cytokine storm

## Abstract

**Background:**

The crucial role of type I interferon (IFN-I, IFN-α/β) is well known to control central nervous system (CNS) neuroinflammation caused by neurotrophic flaviviruses such as Japanese encephalitis virus (JEV) and West Nile virus. However, an in-depth analysis of IFN-I signal-dependent cellular factors that govern CNS-restricted tropism in JEV infection in vivo remains to be elucidated.

**Methods:**

Viral dissemination, tissue tropism, and cytokine production were examined in IFN-I signal-competent and -incompetent mice after JEV inoculation in tissues distal from the CNS such as the footpad. Bone marrow (BM) chimeric models were used for defining hematopoietic and tissue-resident cells in viral dissemination and tissue tropism.

**Results:**

The paradoxical and interesting finding was that IFN-I signaling was essentially required for CNS neuroinflammation following JEV inoculation in distal footpad tissue. IFN-I signal-competent mice died after a prolonged neurological illness, but IFN-I signal-incompetent mice all succumbed without neurological signs. Rather, IFN-I signal-incompetent mice developed hemorrhage-like disease as evidenced by thrombocytopenia, functional injury of the liver and kidney, increased vascular leakage, and excessive cytokine production. This hemorrhage-like disease was closely associated with quick viral dissemination and impaired IFN-I innate responses before invasion of JEV into the CNS. Using bone marrow (BM) chimeric models, we found that intrinsic IFN-I signaling in tissue-resident cells in peripheral organs played a major role in inducing the hemorrhage-like disease because IFN-I signal-incompetent recipients of BM cells from IFN-I signal-competent mice showed enhanced viral dissemination, uncontrolled cytokine production, and increased vascular leakage. IFN-I signal-deficient hepatocytes and enterocytes were permissive to JEV replication with impaired induction of antiviral IFN-stimulated genes, and neuron cells derived from both IFN-I signal-competent and -incompetent mice were vulnerable to JEV replication. Finally, circulating CD11b^+^Ly-6C^+^ monocytes infiltrated into the distal tissues inoculated by JEV participated in quick viral dissemination to peripheral organs of IFN-I signal-incompetent mice at an early stage.

**Conclusion:**

An IFN-I signal-dependent model is proposed to demonstrate how CD11b^+^Ly-6C^+^ monocytes are involved in restricting the tissue tropism of JEV to the CNS.

## Introduction

Flaviviruses are positive-sense, single-stranded RNA viruses transmitted principally by mosquitos and include dengue virus (DenV), Zika virus (ZIKV), Japanese encephalitis virus (JEV), and West Nile virus (WNV) [1]. Among them, JEV is the leading cause of viral encephalitis in Asia and the Western Pacific and is spreading to previously unaffected regions such as Indonesia, Pakistan, and northern Australia [2]. Thus, it is now becoming an increasing threat to global health and welfare, with approximately 67,900 reported cases annually [2]. Japanese encephalitis (JE) is fatal in 25 to 30% of cases, mostly in infants, and a high proportion of patients who survive have serious neurological and psychiatric sequelae [3]. For this reason, JE is considered to be more fatal than WNV encephalitis, resulting in 3–5% mortality (1100 death/29,000 symptomatic infection) [4].

Although more detailed pathogenesis of JE remains to be elucidated, considerable progress has been made in human patients and murine infection models [5–7]. Neuron cells in the CNS are very permissive to JEV replication and are killed directly. In addition, CNS invasion by JEV causes the stimulation of microglia and glia and infiltrated leukocytes, which subsequently leads to indirect neuronal killing via a huge production of pro-inflammatory cytokines (IL-6 and TNF-a) and soluble neurotoxin-like mediators [8, 9]. This suggests that JE is an immunopathological disease caused by uncontrolled over-activation of innate and adaptive immune cells, resulting in neurological disorders in the CNS. The adaptive immune responses, including JEV-specific T cells and virus-neutralizing IgM and IgG, appear to play a role in regulating viral replication in both the peripheral tissues and CNS [10]. However, it is believed that innate immune cells play a more crucial role in the early control of JEV infection than adaptive immunity because adaptive immune responses take time to establish. Various innate immune cells including macrophages [11], dendritic cells (DCs) [7], and NK cells [12, 13] are reported to control JEV replication at an early stage. In addition to their defense role, innate immune cells may also be responsible for generating pathological levels of JEV through uncontrolled over-activation. CNS infiltration and activation of peripheral leukocytes during JE have been known to cause profound damage if the reaction is excessive or inappropriate [14]. Therefore, adequate CNS infiltration and activation of innate immune cells at the periphery is considered to play a critical role in protecting hosts without tissue injury from viral encephalitis such as JE.

The activation of innate immune cells is triggered by recognition of pathogen-associated molecular patterns (PAMPs) produced from invading pathogens through pattern recognition receptors (PRRs) of host cells, such as Toll-like receptors (TLRs), retinoic acid-inducible gene I (RIG-I), and melanoma differentiation-associated protein 5 (MDA5) [15, 16]. Cellular and intracellular activation of innate immune cells and the actions of type I interferon (IFN-I, IFN-α/β) via PRRs provide a first-line defense against virus infection and is known to be an essential modulator in flavivirus replication, dissemination, and neurovirulence [17, 18]. IFN-I is a family of antiviral cytokines that are produced from various infected cells by recognition of flavivirus RNA intermediates through cytosolic and endosomal PRRs, such as RIG-I-like receptors (RLR) or TLRs, which signal through adaptor molecules (e.g., MAVS, TRIF, and MyD88) and transcription factors (e.g., IRF3 and IRF7) [19]. Indeed, the critical role of IFN-I and its transcription factors (IRF3, IRF5, and IRF7) has been described in flavivirus infection in vivo including WNV and DenV, but not JEV [20–22]. IFN-I secreted from infected cells binds in an autocrine and paracrine manner to a heterodimeric IFN-α/β receptor (IFNAR1) on the surface of cells and mediates pleiotropic effects via canonical Janus kinase (JAK)-signal transducer and activator of transcription (STAT1/2) signaling pathway [23, 24]. Phosphorylated STAT1/2 heterodimer associates with IRF-9 to form ISGF3 complexes, which results in the induction of antiviral IFN-stimulated genes (ISGs, e.g., Mx1, OAS, PKR, ISG49 [IFIT3], ISG54 [IFIT2], ISG56 [IFIT1]) through its nuclear translocation and binding to IFN-stimulated responsive elements [19]. The importance of this pathway is underscored by the vulnerability of mice deficient in IFNAR1 and its downstream factors (IFIT1, viperin, Ifi27l2a) to flavivirus infection, mostly WNV infection [25–29]. Similarly, the important role of IFN-I innate immune responses in controlling JEV infection is described in murine infection models using TLRs, 4-1BB, and indoleamine 2,3-dioxygenase (IDO)-deficient mice [30–32]. Conversely, flaviviruses have also evolved various mechanisms for initiating replication in host cells by disrupting the action of IFN-I and evading IFN-I-stimulated antiviral responses [33–36]. Notably, the interplay between JEV and the IFN-I system has been studied in reporter cell lines [34–36]. Collectively, IFN-I innate responses play a critical role in controlling JEV infection and its inhibitory roles in JEV replication have been demonstrated in several studies using reporter cell lines. IFNAR is expressed in all nucleated cells, and individual cell types may respond differentially to signaling after IFN-I binding because distinct transcriptional programs overlap in individual cell subtypes following RNA virus infection [37, 38]. Therefore, although IFN-I innate responses are considered to play an important role in controlling the cell and tissue tropism of WNV and other flaviviruses [17, 18], the IFN-I signal-dependent cellular factors that restrict tissue tropism (e.g., the CNS) in infection models with JEV in vivo are largely unknown.

In the present study, we examined viral dissemination and tissue tropism to the CNS of the host after JEV inoculation in distal tissues such as the footpad using IFN-I-competent (wild type) and -incompetent (IFNAR1 KO) mice. One paradoxical and interesting finding was that IFN-I signaling is essentially required for CNS neuroinflammation following JEV inoculation in the distal footpad tissues because IFN-I signal-incompetent mice all succumbed without any neurological signs. Rather, IFN-I signal-incompetent mice developed a hemorrhage-like disease. This hemorrhage-like disease, which was altered from the typical disease (e.g., encephalitis) of JEV infection, appeared to be driven by quick viral dissemination and impaired IFN-I responses before the invasion of the virus into the CNS. The intrinsic IFN-I signal in tissue-resident cells was critical in limiting the tissue tropism of viral dissemination following distal JEV inoculation, and the early and higher infection of circulating CD11b^+^Ly-6C^+^ monocytes at the inoculation site contributed to viral dissemination into the entire body at the early stage. Therefore, our study proposes a model of IFN-I signal-dependent cellular factors in viral tissue tropism to induce CNS neuroinflammation following distal JEV inoculation.

## Materials and methods

### Ethics statement

All animal experiments described in the present study were conducted at Jeonbuk National University according to the guidelines set by the Institutional Animal Care and Use Committees (IACUC) of Jeonbuk National University and were pre-approved by the Ethical Committee for Animal Experiments of Jeonbuk National University (Permission code 2013–0028). The animal research protocol in this study followed the guideline set up by the nationally recognized Korea Association for Laboratory Animal Sciences (KALAS). The live JEV Beijing-1 strain has been handled in level 2 biosafety laboratory, and the institutional bio-safety committees (IBC) of Jeonbuk National University approved all necessary experimental biosafety protocols in this study.

### Animals, cells, and in vivo viral infection

IFN-I signal-competent C57BL/6 (BL/6, H-2^b^) mice (5–6 weeks old) were purchased from Samtako (O-San, Korea). IFNAR1 knock-out (KO) mice (H-2^b^) deficient in the receptor IFNAR1 for type I IFNs (IFN-α/β) are described elsewhere [39]. The JEV Beijing-1 strain was obtained from the Green Cross Research Institute (Suwon, Korea) and propagated in a mosquito cell line (C6/36) using DMEM supplemented with 2% FBS, penicillin (100 U/ml), and streptomycin (100 U/ml), as described previously [30–32]. The virus stocks were titrated by a focus-forming assay and were stored in aliquots at – 80 °C until use. IFN-I signal-competent (BL/6) and -incompetent (IFNAR1 KO) mice were infected with different doses of JEV (1 × 10^6^, 5 × 10^6^, 1 × 10^7^, and 5 × 10^7^ focus-forming unit (ffu)/mouse) via the intraperitoneal and footpad route for systemic and intradermal infection, respectively.

### Antibodies and reagents

The mAbs used for flow cytometric analysis and other experiments were obtained from eBioscience (San Diego, CA), Biolegend (San Diego, CA), and BD Biosciences (San Diego, CA, USA). These include FITC-conjugated anti-mouse-F4/80 (BM8), PE-conjugated anti-mouse CD11b (M1/70), and PerCP-Cy5.5-conjugated anti-mouse Ly-6C (HK 1.4). The mAb against nonstructural protein 1 (NS1) and E glycoprotein of JEV (JN1 and JE1) was obtained from Abcam (Cambridge, MA), and APC-labeled goat F(ab′)2 anti-mouse IgG (H + L) was obtained from Southern Biotechnology (Birmingham, AL, USA). JEV-specific primers for detecting viral RNA (JEV10,564-10,583 forward, 5′-CCC TCA GAA CCG TCT CGG AA-3′ and JEV10,862-10,886 reverse, 5′-CTA TTC CCA GGT GTC AAT ATG CTG T-3′) [30–32] and primers specific for the cytokines, type I IFNs (IFN-α/β), CC chemokines, RLRs, IRFs, ISGs, tight junction (TJ), and adhesion molecules (Table 1) were synthesized at Bioneer Corp. (Daejeon, Korea) and used for the PCR amplification of target genes.

**Table 1:** Specific primers for the expression of cytokines, chemokines, transcription factor, and JEV RNA used in real-time qRT-PCR

Gene name^a^	Primer sequence (5’-3’)^b^	Position cDNA	Gene bank ID
TNF-α	FP: CGT CGT AGC AAA CCA CCA AG RP: TTG AAG AGA ACC TGG GAG TAG ACA	449-468 575-598	NM_013693.3
IL-6	FP: TGG GAA ATC GTG GAA ATG AG RP: CTC TGA AGG ACT CTG GCT TTG	256-275 489-509	NM_031168.2
IFN-α	FP: TGT CTG ATG CAG CAG GTG G RP: AAG ACA GGG CTC TCC AGA C	367-385 514-532	NM_008334.3
IFN-β	FP: TCC AAG AAA GGA CGA ACA TTC G RP: TGA GGA CAT CTC CCA CGT CAA	106-127 399-419	NM_010510.1
CCL2	FP: AAA AAC CTG GAT CGG AAC CAA RP: CGG GTC AAC TTC ACA TTC AAA G	347-367 426-447	NM_011333
CXCL2	FP: ATC CAG AGC TTG AGT GTG ACG C RP: AAG GCA AAC TTT TTG ACC GCC	194-215 263-283	NM_009140.2
RIG-I	FP: CCA CCT ACA TCC TCA GCT ACA TGA RP: TGG GCC CTT GTT GTT CTT CT	194-217 260-279	NM_172689.3
MDA5	FP: GGC ACC ATG GGA AGT GAT T RP: ATT TGG TAA GGC CTG AGC TG	1178-1196 1247-1266	NM_027835.3
IRF3	FP: GAT GGA GAG GTC CAC AAG GA RP: GAG TGT AGC GTG GGG AGT GT	1170-1189 1259-1278	NM_016849.4
IRF7	FP: CCT CTT GCT TCA GGT TCT GC RP: GCT GCA TAG GGT TCC TCG TA	980-999 1080-1099	NM_016850.3
ISG49	FP: GCC GTT ACA GGG AAA TAC TGG RP: CCT CAA CAT CGG GGC TCT	919-939 1126-1143	NM_010501.2
ISG54	FP: GGG AAA GCA GAG GAA ATC AA RP: TGA AAG TTG CCA TAC AGA AG	1434-1453 1587-1606	NM_008332.3
ISG56	FP: CAG AAG CAC ACA TTG AAG AA RP: TGT AAG TAG CCA GAG GAA GG	774-793 911-930	NM_008331.3
Claudin-1	FP: TCT ACG AGG GAC TGT GGA TG RP: TCA GAT TCA GCA AGG AGT CG	350-369 414-433	NM 016674.4
Claudin-5	FP: GTG GAA CGC TCA GAT TTC AT RP: TGG ACA TTA AGG CAG CAT CT	1054-1073 1131-1150	NM 013805.4
Occludin	FP: GCT GTG ATG TGT GTG AGC TG RP: GAC GGT CTA CCT GGA GGA AC	2054-2074 2105-2124	NM 008756.2
ZO-1	FP: AGG ACA CCA AAG CAT GTG AG RP: GGC ATT CCT GCT GGT TAC A	6227-6246 6295-6313	NM 009386.2
ICAM-1	FP: GTC CGC TGT GCT TTG AGA ACT RP: CGG AAA CGA ATA CAC GGT GAT	358-378 411-431	NM 010493.3
JAM	FP: ACC CTC CCT CCT TTC CTT AC RP: CTA GGA CTC TTG CCC AAT CC	1163-1182 1238-1257	NM 172647.2
β-actin	FP: TGG AAT CCT GTG GGA TCC ATG AAA C RP: TAA AAC GCA GCT CAG TAA CAG TCC G	915-939 1239-1263	NM_007393.5

^a^ *IL* interleukin

^b^ *FP* forward primer, *RP* reverse primer

### Quantitation for viral burden, cytokine, TJs, and adhesion molecule expression

#### Quantitative real-time PCR for viral burden, cytokine, TJ, and adhesion molecule mRNA

Viral burden, cytokine (IL-6, TNF-α, IFN-α/β), chemokines (CCL2, CXCL2), RLRs (RIG-1, MDA5), IRFs (IRF3, IRF7), ISGs (ISG49, ISG54, ISG56), TJs (Claudin-1, Claudin-5, Occludin, ZO-1), and adhesion molecules (ICAM-1, JAM) were determined by conducting quantitative SYBR green-based real-time RT PCR (real-time qRT-PCR). IFN-I signal-competent and -incompetent mice were infected with JEV (5 × 10^6^ ffu/mouse) via the footpad route, and the non-lymphoid and lymphoid tissues including popliteal lymph nodes (pLNs), spleen, mesenteric LNs (mLNs), bone marrow (BM), iliac LNs (iLNs), lung, liver, intestine, kidney, spinal cord (SC), and brain were harvested at 1, 2, and 3 dpi. Total RNAs extracted from collected tissues using Wizol^TM^ reagent (Wizbiosolutions Inc, Seongnam, Korea) were used for real-time qRT-PCR using a CFX96 Real-Time PCR Detection System (Bio-Rad Laboratories, Hercules, CA, USA). The reverse transcription of total RNAs with High-Capacity cDNA Reverse Transcription Kits (Applied Biosystems, Foster, CA) was processed before real-time qPCR. The reaction mixture contained 2 μl of template cDNA, 10 μl of 2× SYBR Primix Ex Taq, and 200 nM specific primers at a final volume of 20 μl. The reaction mixes were denatured at 95 °C for 30 s and then subjected to 45 cycles of 95 °C for 5 s and 60 °C for 20 s. After the reaction cycle was completed, the temperature was increased from 65 to 95 °C at a rate of 0.2 °C/15 s, and the fluorescence was measured every 5 s to construct a melting curve. A control sample containing no template cDNA was run with each assay, and all determinations were performed at least in duplicate to ensure reproducibility. The authenticity of the amplified product was determined by melting curve analysis. The viral burden was expressed as viral RNA copy number per microgram of total RNA, and the expression of cytokines, chemokines, RLRs, IRFs, ISGs, TJs, and adhesion molecules was presented as the relative fold expression after normalization to the housekeeping gene β-actin. All data were analyzed using Bio-Rad CFX Manager, version 2.1 analysis software (Bio-Rad Laboratories).

#### Focus-forming assay (FFA) for infectious JEV

The amount of infectious JEV in tissues and culture media was measured by FFA. Briefly, the FFA was performed by incubating 4-fold serially diluted virus (from 1:4 to 1:64) with Vero cells (around 80% confluent) in 24-well plates. After 1 h at 37 °C, the wells were overlaid with 0.5 ml 0.8% methylcellulose (Thermo Fisher Scientific, Inc., Waltham, MA) in Opti-MEM Media (Invitrogen Corp., Carlsbad, CA) containing 2% FBS. Following a 3-day incubation, the methylcellulose overlay was removed, and the cells in each well were washed with PBS, followed by fixation with cold MeOH. The cells were blocked, permeabilized with 10% goat serum containing 0.5% TritonX100 for 1 h at room temperature, followed by washing twice with PBS. Subsequently, the cells were stained intracellularly with mAb specific for JEV NS1 protein (Abcam, Cambridge, UK) for 90 min, followed by staining with HRP-conjugated goat anti-mouse IgG (H + L) (Southern Biotech). The focus-forming spots were visualized using WEST-ZOL Plus (iNtRON, Daejeon, Korea) in Fusion FX7 (Vilber Lourmat, Collegien, France), according to the manufacturer’s protocol. Photographs were taken on the same day as staining. The ffu/culture media or tissue was calculated by counting focus-forming spots.

#### Cytokine ELISA

A sandwich ELISA was used to determine the levels of IL-6 and TNF-α cytokines in sera of IFN-I signal-competent and -incompetent mice following JEV infection. ELISA plates were coated with IL-6 (MP5-20F3), TNF-α (1F3F3D4) anti-mouse Ab purchased from eBioscience, and then incubated overnight at 4 °C. The plates were washed three times with PBS containing 0.05% Tween (PBST), after which they were blocked with 3% bovine serum albumin for 2 h at 37 °C. The sera and standards for recombinant cytokine proteins (PeproTech, Rocky Hill, NJ) were added to the plates, which were then incubated for 2 h at 37 °C. The plates were washed again with PBST, and biotinylated IL-6 (MP5-32C11) and TNF-α (MP6-XT22) Ab were added. Next, the plates were incubated overnight at 4 °C, followed by washing with PBST and subsequent incubation with peroxidase-conjugated streptavidin (eBioscience) for 1 h. Color development was then performed by the addition of a substrate (ABTS) solution. Cytokine concentrations were determined with an automated ELISA reader and SoftMax Pro3.4 according to comparisons with two concentrations of standard cytokine proteins.

#### Cytometric bead array (CBA) for CC chemokine production

The level of CC chemokine proteins in sera was measured by a CBA specific for CCL2 (MCP-1), CCL3 (MIP-1α), CCL4 (MIP-1β), CCL5 (RANTES), and CCL7 (MCP-3), according to the manufacturer’s protocols (BD Bioscience).

### Complete blood count (CBC) and serum biochemistry

CBC and serum biochemistry were assessed as described previously [40]. For CBC data, whole blood collected from IFN-I signal-competent and -incompetent mice was treated with K2EDTA to prevent clotting, and samples were immediately analyzed using an automated hematology analyzer (Nihon Kohden, Tokyo, Japan), according to the manufacturer’s instructions. For the functional evaluation of liver and kidney organs, blood was collected to obtain plasma and immediately analyzed using a clinical chemistry analyzer (Mindray, Shenzhen, China) according to the manufacturer’s instructions.

### Evaluation of vascular permeability

Vascular leakage in IFN-I signal-competent and -incompetent mice was measured following JEV infection, as described elsewhere [30]. Briefly, 0.2 ml of Evans blue dye (0.5% solution in PBS) was injected into infected mice by the intravenous route at 48 h pi. After 2 h, the mice were extensively perfused with PBS via cardiac puncture of the left ventricle. Samples of liver and intestine were then harvested, and vascular permeability was evaluated by visualizing collected organs. The amount of Evans blue dye extravasated into tissues was measured by a colorimetric method. After weighing, the tissues were homogenized in 2 ml of PBS and 2 ml of 100% trichloroacetic acid (TCA; Sigma-Aldrich, St Louis, MO) was added into the homogenate. Then the mixture was vigorously shaken for 5 min and cooled for 40 min at 4 °C. After centrifugation (40 min at 800 × g), the absorbance of the supernatant was measured at 620 nm using a spectrophotometer. Data were expressed as micrograms of Evans blue dye per gram of organ tissue using a standard curve.

### Histopathological examinations and immunohistochemistry

Histopathological examination was performed using various non-lymphoid and lymphoid tissues including popliteal LNs (pLNs), spleen, mesenteric LNs (mLNs), liver, intestine, and brains derived from IFN-I signal-competent and -incompetent mice following JEV infection via the footpad route. Tissues were embedded in paraffin at 2 dpi, and 10-μm sections were prepared and stained with H&E. Following deparaffinization, tissue sections were also used for the detection of JEV Ags by staining with mAbs specific for E and/or NS1 proteins. After antigen retrieval, endogenous peroxidases were quenched by incubating the slides in 3% H_2_O_2_ for 15 min. The sections were then washed with PBS for 10 min. Endogenous avidin and biotin were blocked using a SuperBlock blocking buffer according to manufacturer’s instructions (Thermo Fisher Scientific, Inc.). The sections were then washed with PBS for 4 min. Primary antibodies specific for JEV E and/or NS1 proteins were applied overnight at 4 °C in a humidified chamber. After rinsing the slides in PBS, they were incubated in secondary antibody for 30 min at room temperature. After washing with PBS for 5 min, color development was achieved by applying diaminobenzidine tetrahydrochloride (DAB) solution (Vector Laboratories) for 0.5–1 min. After washing in distilled water, the sections were counterstained with VECTOR methyl Green (Vector Laboratories), and cover-slipped using a mounting medium (Thermo Fisher Scientific). Sections were analyzed using a Nikon Eclipse E600 microscope (Nikon, Tokyo, Japan).

### Generation of BM chimeric mice

C57BL/6 (BL/6) and IFNAR1 KO mice were γ-irradiated with one dose of 950 rads. Within 12 h, the stem cells of recipient mice were reconstituted with BM cells (1.5 × 10^7^ cells/mouse) from BL/6 and IFNAR1 KO donor mice, respectively. Sulfamethoxazole and trimethoprim were added to the drinking water of recipient mice for the next 10 days. Recipient mice were allowed to reconstitute their hematopoietic stem cell (HSC) population for 4 to 6 weeks. Chimerism was confirmed before experimental use. BM chimeric mice were infected with JEV (5 × 10^6^ ffu/mouse) via the footpad. The infected recipients were monitored daily for survival and neurological disorders.

### Primary hepatocyte, enterocyte, and cortical neuron cells

#### Primary hepatocyte

Primary hepatocytes were isolated from IFN-I signal-competent and -incompetent mice using a modified two-step collagenase perfusion method [30]. Mouse primary hepatocytes were then cultured using type I rat tail collagen-coated 12-well plates in William’s E medium containing 10% FBS, 100 U/ml penicillin, and 100 μg/ml streptomycin at 37 °C in a humidified incubator. At 70–80% confluence, the primary hepatocytes were infected with JEV at different multiplicity of infection (MOI; 0.01, 0.1, and 1.0).

#### Primary enterocytes

Primary enterocytes were prepared using a modified method, as described elsewhere [41, 42]. Small intestines were opened longitudinally and cut into small pieces (~ 1 mm × 1 mm). After being washed, the tissues were soaked on ice for 90 min in Ca^2+^ and Mg^2+^-free Hank’s balanced salt solution (HBSS) (Thermo Fisher Scientific, Inc.) containing 3 mM EDTA and 1 mM DTT. The tissues were shaken vigorously to detach crypts, which were pelleted by centrifugation at 150 × g for 3 min at 4 °C. The pellets were washed twice with ice-cold Ca^2+^ and Mg^2+^-free HBSS and resuspended in DMEM/F12 culture medium (BioWhittaker, Walkersville, MD), which was supplemented with 10% FBS, 100 U/ml penicillin, 100 μg/ml streptomycin, 1 × insulin-transferrin-selenium (Invitrogen), 20 mM HEPES (pH 7.4), 0.25 μg/ml amphotericin B, 1 μg/ml fibronectin, and 1 μg/ml hydrocortisone. Twenty-four-well plates were coated with collagen I and poly-L-lysine 1 h before the cells were seeded. The plates were seeded with approximately 200 to 300 organoids/cm^2^ and incubated at 37 °C under a 5% CO_2_ atmosphere. Three days after being seeded, the cells were infected with JEV at different MOI (0.01, 0.1, and 1.0).

#### Primary cortical neuron cells

Primary cortical neurons were prepared from 15-day-old embryos as described previously [43]. Briefly, ganglia were treated with collagenase (1 mg/ml) for 30 min at 37 °C followed by 2.5 mg/ml trypsin for 30 min at 37 °C. The ganglia were then triturated with a flame-polished Pasteur pipette and filtered through a 3-20/14 Nitex filter (Tetko Inc., Elmsford, NY). Cell viability was assessed by trypan blue exclusion. Cortical neurons were seeded in 12-well poly-D-lysine/laminin-coated plates in DMEM containing 5% FBS and 5% horse serum for 24 h, and then cultured for 4 days with neurobasal medium containing B27 supplement and L-glutamine (Invitrogen, Carlsbad, CA). Primary cortical neurons were infected with JEV at different MOI (0.01, 0.1, 1.0).

### In vivo detection of JEV-infected myeloid-derived cells

JEV-infected myeloid-derived cells were detected by intracellular staining for JEV NS1 Ag using cells prepared from the footpad, liver, blood, and brain. Briefly, infiltrated cells in the footpad were prepared from IFN-I signal-competent and -incompetent mice 1 and 2 dpi by collagenase digestion of footpad tissues, and total cells including hepatocytes and infiltrated leukocytes were obtained from the liver of the mice using a modified two-step collagenase perfusion method. Peripheral blood leukocytes were also prepared by treating blood cell suspension with ammonium chloride-containing Tris buffer (NH_4_Cl-Tris) for 5 min at 37 °C. Infiltrated leukocytes in the brain were prepared by collagenase digestion following vigorous perfusion via cardiac puncture of the left ventricle [30–32]. Cells prepared from the footpad, liver, blood, and brain were surface-stained with an antibody cocktail for CD11b, F4/80, and Ly-6C, and then intracellularly stained with mAb specific for JEV NS1 protein (JN1, Abcam), followed by APC-labeled goat (ab′)2 anti-mouse IgG (H + L) (Southern Biotechnology) for 30 min at 4 °C. Finally, the cells were washed twice with PBS and fixed using fixation buffer. Sample analysis was performed with a FACS Calibur flow cytometer (Becton Dickson Medical Systems) and FlowJo (Tree Star) software.

### Statistical analysis

All data were expressed as average ± standard error of the mean (SEM). Statistically significant differences between groups were analyzed using an unpaired two-tailed Student’s *t* test for ex vivo experiments and immune cell analysis. For multiple comparisons, statistical significance was determined using one-way or two-way analysis of variance (ANOVA) with repeated measures, followed by Bonferroni post hoc tests. Kaplan–Meier survival curves were analyzed by the log-rank test. A p value ≤ 0.05 was considered significant. All data were analyzed using GraphPadPrism4 software (GraphPad Software, Inc., San Diego, CA).

## Results

### IFN-I signaling is required for CNS neuroinflammation following JEV inoculation in distal tissues

**Fig. 1:**
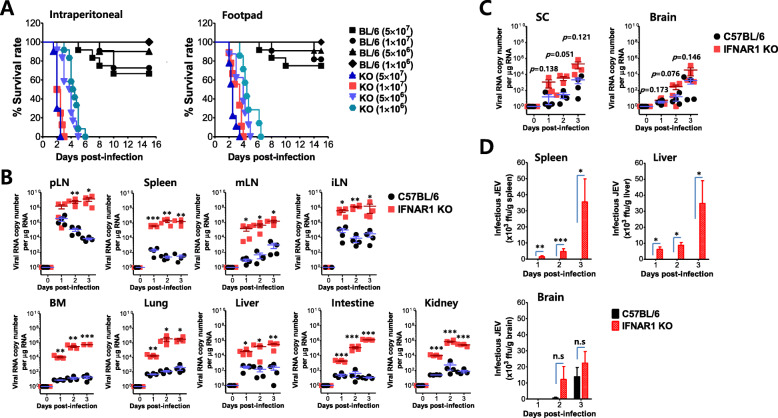
IFN-I signaling is an indispensable requirement in CNS neuroinflammation following distal JEV inoculation. **a** Essential role of IFN-I signaling in providing protection from JE. The susceptibility of C57BL/6 (BL/6) and IFNAR1 KO mice (*n* = 8–12) to JEV infection were monitored following different doses of viral inoculation (1 × 10^6^, 5 × 10^6^, 1 × 10^7^, and 5 × 10^7^ ffu/mouse) via intraperitoneal and footpad routes. The survival rate was examined over 15 days. **b** Viral burden in the non-lymphoid and lymphoid tissues at the periphery. Following JEV infection (5 × 10^6^ ffu/mouse) via the footpad route, viral burden was assessed by real-time qRT-PCR at the indicated days pi in various tissues including popliteal LNs (pLNs), spleen, mesenteric LNs (mLNs), iliac LNs (iLN), bone marrow (BM), lung, liver, intestine, and kidney. **c** Viral burden in the CNS. Viral burden in the CNS tissues including spinal cord (SC) and brain was determined by real-time qRT-PCR using total RNA extracted from tissues. The viral RNA load was expressed by JEV RNA copy number per microgram of total RNA (*n* = 4). Each symbol represents the level in an individual mouse; the horizontal line indicates the mean ± SEM of each group. **d** Infectious JEV burden in both peripheral and CNS tissues. Following JEV infection (5 × 10^6^ ffu/mouse) via the footpad route, the amount of infectious JEV was determined by a focus-forming assay using PBS-homogenates of the indicated tissues. Data in the graphs denote the mean ± SEM. Results are representative of one out of at least two individual experiments with four to five mice per group. Statistical significance *p < 0.05; **p < 0.01; ***p < 0.001 was assessed by an unpaired two-tailed Student’s *t* test

IFN-I signaling has been known to play a crucial role in conferring protection against various viral infections [20–22]. However, how IFN-I participates in viral dissemination and CNS neuroinflammation following JEV infection remains largely unknown. To test whether IFN-I signaling plays a role in inducing neurological disorders (JE) following JEV infection, IFN-I signal-competent (BL/6) and -incompetent (IFNAR1 KO) mice were infected with JEV at different doses (1 × 10^6^, 5 × 10^6^, 1 × 10^7^, and 5 × 10^7^ ffu) via the intraperitoneal or footpad route for systemic or local infection, respectively. As expected, IFN-I signal-incompetent mice show highly enhanced susceptibility to JEV infection, compared with IFN-I signal-competent mice (Fig. 1a). In contrast with IFN-I signal-competent mice, incompetent mice all succumbed to JEV infection within 6 and 7 days following JEV infection at a low dose (1 × 10^6^ ffu/mouse) via the intraperitoneal and footpad route, respectively. Here, the interesting result was that IFN-I signal-competent mice infected with JEV (5 × 10^7^ ffu/mouse) via the footpad route died through a prolonged and neurological illness that was symbolized by weight loss, distressed fur, and back hunching with postural imbalance, ataxia, and generalized tonic-clonic seizure, whereas IFN-I signal-incompetent mice all died without any neurological signs following JEV infection at different doses via the footpad route (Table 2). This result implies that while IFN-I signal-competent mice show typical JE signs of CNS neuroinflammation after JEV inoculation at the distal site, IFN-I signal-incompetent mice succumb to other fatal damages. To better understand fatal damages in IFN-I signal-incompetent mice following JEV infection, we determined the viral burden in various peripheral lymphoid and non-lymphoid tissues, as well as CNS tissues such as the brain and SC. IFN-I signal-incompetent mice were found to contain 10^2^–10^4^-fold increased levels of viral RNA in peripheral lymphoid and non-lymphoid tissues, including pLNs, spleen, mLNs, iliac LNs, BM, lung, liver, intestine, and kidney, 1, 2, and 3 days after JEV inoculation at footpad tissues, compared with IFN-I signal-competent mice (Fig. 1b). In contrast with peripheral lymphoid and non-lymphoid tissues, IFN-I signal-incompetent mice showed no significant increase in the levels of viral RNA in the SC and brain, even though they contained modestly increased viral loads in CNS tissues compared with competent mice (Fig. 1c). In addition, infectious JEV was recovered at higher levels from peripheral tissues such as the spleen and liver in IFN-I signal-incompetent mice than in the competent mice (Fig. 1d). However, infectious JEV was detected at levels with no significant increase in the brain of IFN-I signal-incompetent mice, compared with IFN-I signal-competent mice. These data indicate that IFN-I signaling plays a crucial role in regulating viral dissemination to peripheral tissues at the early stage after the local inoculation of JEV. Conversely, our data also suggest that IFN-I signal is required to induce neurological disorders like JE as CNS neuroinflammation at the late stage (around 7−9 days) after JEV inoculation at distal sites such as the footpad.

**Table 2:** The number of mice showing neurological diseases following JEV infection

		Intraperitoneal			Footpad		
Strain	Dose (pfu)	d7	d8	d9	d7	d8	d9
BL/6	5 × 10^7^	2/12	2/12	3/12	1/12	2/11	3/12
	1 × 10^7^	1/11	2/11	3/11	0/11	0/11	2/11
	5 × 10^6^	1/11	1/11	1/11	0/11	1/11	1/11
	1 × 10^6^	0/11	0/11	0/11	0/11	0/11	0/11
KO	5 × 10^7^	Not found			Not found		
	1 × 10^7^						
	5 × 10^6^						
	1 × 10^6^						

### Requirement of IFN-I signaling for CNS neuroinflammation through the restriction of JEV dissemination

**Fig. 2:**
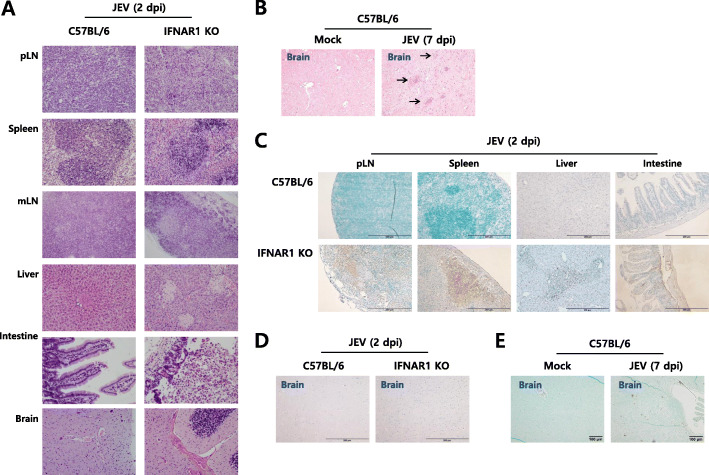
IFN-I signal limits the pathological process in non-lymphoid and lymphoid peripheral tissues. **a** Histopathological examinations of the peripheral and CNS tissues in IFN-I signal-competent and -incompetent mice. Following JEV infection (5 × 10^6^ ffu) via the footpad route, paraffin-embedded tissues including popliteal LNs (pLNs), spleen, mesenteric LNs (mLNs), liver, intestine, and brain were prepared on the 2nd day, and used for histological examinations with H&E staining. **b** Histopathological examinations of the CNS in IFN-I signal-competent mice showing neurological disorders following JEV infection. The arrows denote the area of interest. **c** Detection of JEV Ags in the non-lymphoid and lymphoid tissues in the periphery of IFN-I incompetent mice. **d** Detection of JEV Ags in the brain of both IFN-I signal-competent and -incompetent mice. **e** Detection of JEV Ags in the CNS in IFN-I signal-competent mice showing neurological disorders following JEV infection. Sections prepared from lymphoid and non-lymphoid tissues including pLNs, spleen, liver, intestine, and brain were used for the detection of JEV Ags (NS1 and E proteins). Images are representative of sections (200×) derived from at least four mice. Areas of interest infiltrated with leukocytes are denoted by black arrows

To understand fatal damages and viral dissemination in IFN-I signal-incompetent mice following JEV infection, we performed several histopathological examinations and immunohistochemical studies for JEV Ags in various tissues including peripheral lymphoid and non-lymphoid tissues as well as CNS tissue such as the brain. Histopathological examinations revealed that IFN-I signal-incompetent mice had a destructive architecture in peripheral lymphoid tissues, such as pLNs, spleen, and mLNs, 2 days after the footpad inoculation of JEV (Fig. 2a). Notably, the spleen of IFN-I signal-incompetent mice showed reduced white pulp along with the depletion of immune cells, and the size of pLNs and mLNs was considerably reduced in IFN-I signal-incompetent mice. In addition, multifocal necrosis is observed in the whole liver of IFN-I signal-incompetent mice, along with dying cells accompanied by epithelial cells of the villi falling away in the intestine. In contrast with the peripheral lymphoid and non-lymphoid tissues, in both IFN-I signal-competent and -incompetent mice, there was no apparent change in pathological phenomena in the CNS tissue 2 dpi, except that some hemorrhages were observed in the brain stem and adjacent parts of the cerebellum in IFN-I signal-incompetent mice. However, in histopathological examinations of IFN-I signal-competent mice at 6−7 dpi, we could find CNS neuroinflammatory reactions in brain tissues, such as some leukocyte infiltration (Fig. 2b). In addition, JEV Ags were more apparently detected in peripheral lymphoid and non-lymphoid tissues of IFN-I signal-incompetent mice compared with competent mice (Fig. 2c). We observed no significant JEV Ags in both the brains of IFN-I signal-competent and -incompetent mice at 2 dpi (Fig. 2d). However, JEV Ags were detected in the brain of IFN-I signal-competent mice showing neurological disorders (Fig. 2e), which indicates that the JEV invasion of the CNS could be important in mice displaying neurological disorder. Taken together, these histopathological and immunohistochemical examinations support a crucial role of IFN-I signaling in eliciting CNS neuroinflammation by restricting viral dissemination to peripheral tissues following the local inoculation of JEV.

### IFN-I signaling restricts hemorrhage-like diseases following JEV infection

**Fig. 3:**
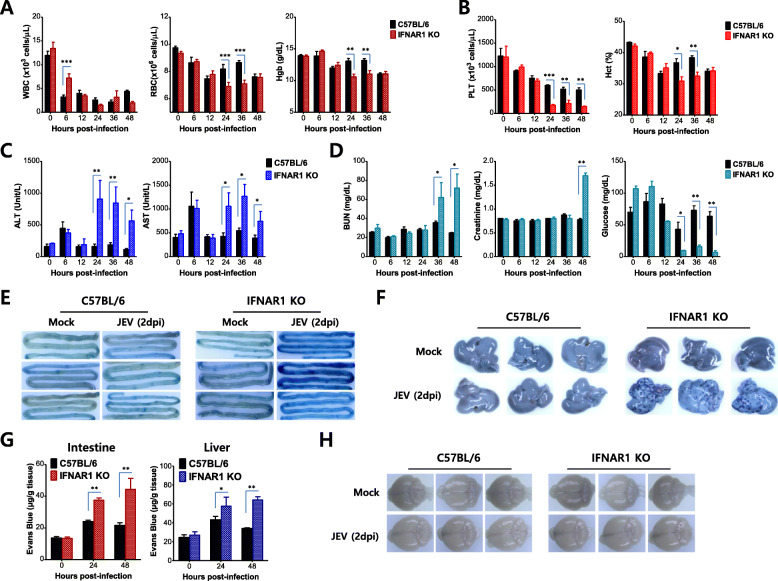
Blood chemistry revealed hemorrhage-like disease in IFN-I signal-incompetent mice rather than CNS neuroinflammation. **a** Complete blood count (CBC) data. The levels of white blood cells (WBC), red blood cells (RBC), and hemoglobin (Hgb) were determined using an automated hematology analyzer at the indicated time points after JEV infection (5 × 10^6^ ffu/mouse) via the footpad route. **b** Platelet number and hematocrit. Platelet (PLT) number and hematocrits (Hct) were measured by an automated hematology analyzer and Hct was expressed as % volume occupied by RBCs. **c** and **d** Injury of liver and kidney function. Liver and kidney function was evaluated by the levels of ALT and AST in the liver **(c)**, and the levels of BUN, creatinine, and glucose in kidneys **(d)** using a clinical chemistry analyzer. **e** and **f** Picture of extravasated Evans blue dye. Two days after JEV infection (5 × 10^6^ ffu/mouse) via the footpad route, IFN-I-competent and -incompetent mice were given 0.5% Evans blue dye solution via a tail vein. Evans blue dye extravasated into the intestine (**e**) and liver (**f)** tissues was visualized following vigorous heart perfusion. **g** The amount of extravasated Evans blue dye into the intestine and liver tissues. The amount of extravasated Evans blue dye was quantified by measuring the absorbance (620 nm) after homogenization and TCA-precipitation of the indicated tissues. **h** Picture of extravasated Evans blue dye into the brain. The extravasated Evans blue dye into the brain was visualized following vigorous heart perfusion. Data in bar graphs denote the mean ± SEM. Results are representative of one out of at least two individual experiments with four to five mice per group. Statistical significance *p < 0.05; **p < 0.01; ***p < 0.001 was assessed by an unpaired two-tailed Student’s *t* test

IFN-I signal-incompetent mice displayed viral dissemination in various peripheral organs and died without CNS neuroinflammation after distal JEV inoculation. Subsequently, we decided to further explore why IFN-I signal-incompetent mice succumbed so rapidly to JEV infection without CNS neuroinflammation. To this end, we analyzed their blood chemistries. The levels of red blood cells (RBC) and hemoglobin were found to significantly drop in IFN-I signal-incompetent mice at 24 and 36 h pi, however, white blood cell (WBC) levels were temporarily higher at an earlier time point of 6 h pi in IFN-I signal-incompetent mice compared with IFN-I signal-competent mice (Fig. 3a). In particular, the number of blood platelets in IFN-I signal-incompetent mice was persistently and markedly decreased from 24 h pi, which indicates that JEV infection caused thrombocytopenia in these mice (Fig. 3b). As a result, IFN-I signal-incompetent mice had lower hematocrit values at 24 and 36 h pi compared with competent mice. Because thrombocytopenia and hematocrit changes may be associated with the damage of major visceral organs [44, 45], we checked liver and kidney functions. Serum alanine aminotransferase (ALT) and aspartate aminotransferase (AST) levels, which are typical parameters for liver function, were at around 5-fold increased levels from 24 h pi in IFN-I signal-incompetent mice compared with competent mice (Fig. 3c). These increases in ALT and AST levels were very correlated with the occurrence of thrombocytopenia at 24 h pi. Similarly, the impaired parameters for kidney function were observed in IFN-I signal-incompetent mice, as shown by increases in blood urea nitrogen (BUN) and creatinine level in sera (Fig. 3d, *left and middle graphs*). The reduction of glucose level in sera of IFN-I signal-incompetent mice may also reflect impaired kidney function (Fig. 3d, *right graph*) because hypoglycemia is associated with renal failure [46], increase in glucose use [47, 48], and decreasing hepatic glucose production [47, 49]. The presence of thrombocytopenia and the damage of major viscera organs such as liver and kidney with fatal non-neurological disease suggested that the inoculation of JEV into the footpad might cause hemorrhage-like disease in IFN-I signal-incompetent mice [50–52]. Vascular leakage is a hallmark of hemorrhagic disease such as the severe form (dengue hemorrhagic fever, DHF) caused by dengue virus infection [50, 51]. Therefore, we checked whether the footpad inoculation of JEV could induce increased vascular permeability in IFN-I signal-incompetent mice. To assess the integrity of the vascular endothelial barrier, IFN-I signal-competent and -incompetent mice were intravenously injected with Evans blue, a dye that preferentially binds to proteins such as albumin. Then, the extravasation of Evans blue dye into tissues was visualized in the intestine and liver following vigorous heart perfusion (Fig. 3e, f). As expected, IFN-I signal-incompetent mice showed increased extravasation of Evans blue dye, as shown by a greater intensity of dark blue in the intestine and liver. Notably, dispersed spots of Evans blue dye occurred in the entire liver tissues of IFN-I signal-incompetent mice, which indicates necrosis by JEV replication. Furthermore, we quantified the amount of Evans blue dye extravasated into liver and intestine tissues spectrophotometrically (Fig. 3g). Similarly, intestine and liver tissues of IFN-I signal-incompetent mice were observed to contain higher levels of extravasated Evans blue dye compared with competent mice. However, dissimilar to the results derived from intestine and liver tissues, brain tissue derived from IFN-I signal-competent and -incompetent mice showed no significantly altered extravasation of Evans blue dye. Collectively, these results support the conclusion that impairment of IFN-I signaling by the distal inoculation of JEV, such as via the footpad route, drives hemorrhagic-like diseases rather than neurological diseases.

### Altered expression of TJs and adhesion molecules results in increased vascular leakage in IFN-I signal-incompetent mice

**Fig. 4:**
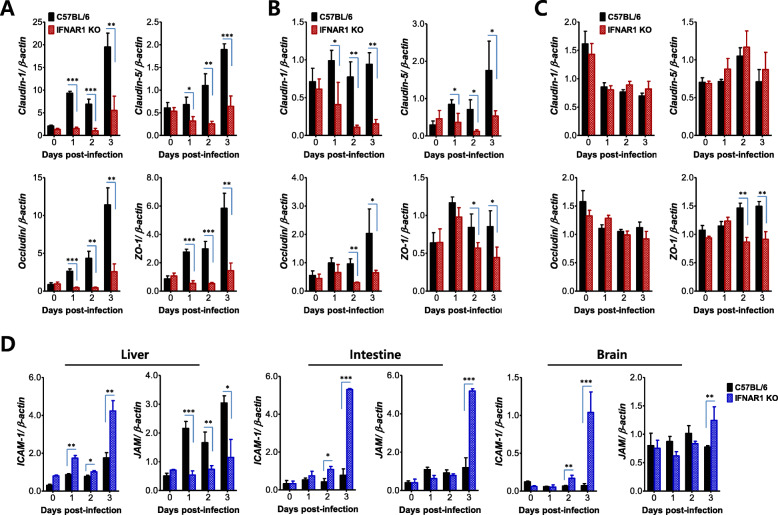
Altered expression of tight junction (TJ) and adhesion molecules in the non-lymphoid peripheral tissues of IFN-I signal-incompetent mice. **a–c** The expression of TJ molecules in peripheral organs and brain of IFN-I signal-competent and -incompetent mice. The mRNA expression of TJ molecules in the liver (**a**), intestine (**b**), and brain (**c**) was evaluated by real-time qRT-PCR at the indicated days after JEV infection (5 × 10^6^ ffu/mouse) via the footpad route. **d** The altered expression of adhesion molecules in IFN-I signal-incompetent mice. The mRNA levels of adhesion molecule including ICAM-1 and JAM-1 were determined by real-time qRT-PCR using total RNA extracted from liver, intestine, and brain at the indicated dpi. Data in bar graphs denote the mean ± SEM. Results are representative of one out of at least two individual experiments with four to five mice per group. Statistical significance *p < 0.05; **p < 0.01; ***p < 0.001 was assessed by an unpaired two-tailed Student’s *t* test

The importance of TJs and adhesion molecules in modulating the invasion of non-neural tissues after JEV infection has been described [53]. Therefore, to further understand the increase of vascular leakage in the peripheral tissue of IFN-I signal-incompetent mice following peripheral JEV inoculation, we checked the expression of TJs and adhesion molecules. The mRNA levels of TJs including claudin-1, claudin-5, occludin, and ZO-1 were significantly reduced in the liver and intestine of IFN-I signal-incompetent mice 2 days following footpad inoculation of JEV (Fig. 4a, b), but mRNA levels of TJs, except ZO-1, in the brain tissue were almost the same in both IFN-I signal-competent and -incompetent mice (Fig. 4c). The expression of the adhesion molecules (ICAM-1 and JAM) in the liver, intestine, and brain appeared complicated. ICAM-1 expression was observed to be higher in the liver and intestine of IFN-I signal-incompetent mice compared with competent mice. However, JAM expression was reduced in the liver of IFN-I signal-incompetent mice but increased in the intestine compared with competent mice (Fig. 4d). Also, ICAM-1 expression was increased in the brain of IFN-I signal-incompetent mice but appeared to be delayed compared with other organs. These results indicate that the expression of adhesion molecules could be differentially regulated in peripheral organs and the CNS, depending on the progression of the disease. Conclusively, no increased expression of TJs in the liver and intestine tissues of IFN-I signal-incompetent mice supports an increase of vascular leakage in peripheral organs, and the altered expression of adhesion molecules in peripheral organs and brain may affect infiltration of leukocytes in inflamed tissues.

### IFN-I signaling restrains the “cytokine storm” following JEV infection

**Fig. 5:**
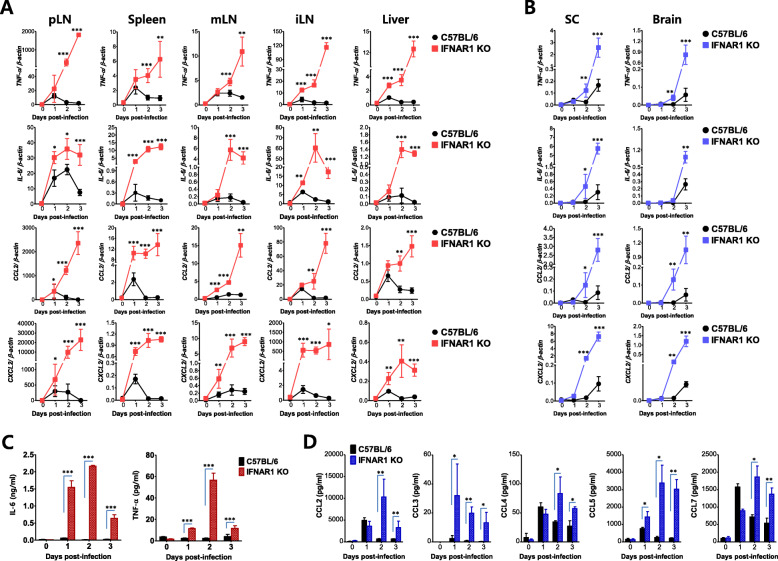
IFN-I signal-dependent induction of a “cytokine storm” following JEV inoculation. **a** and **b** Highly enhanced expression of inflammatory cytokines and chemokines in the peripheral and CNS tissues of IFN-I signal-incompetent mice. The mRNA levels of inflammatory cytokines and chemokines were determined by real-time qRT-PCR using total RNA extracted from popliteal LNs (pLNs), spleen, mesenteric LNs (mLNs), iliac LN (iLN), liver, spinal cord (SC), and brain at the indicated days after JEV infection (5 × 10^6^ ffu/mouse) via the footpad route. **c** Serum levels of IL-6 and TNF-a. **d** Enhanced production of CC chemokines in sera of IFN-I signal-incompetent mice. Serum levels of IL-6 and TNF-α were determined by cytokine ELISA using sera collected from IFN-I signal-competent and -incompetent mice. The levels of CC chemokines including CCL2, CCL3, CCL4, CCL5, and CCL7 in sera were determined by cytokine bead array. Data in the graphs denote the mean ± SEM. Results are representative of one out of at least two individual experiments with four to five mice per group. Statistical significance *p < 0.05; **p < 0.01; ***p < 0.001 was assessed by an unpaired two-tailed Student’s *t* test

To further understand the basis of peripheral organ damage, such as that in the liver and kidney, in IFN-I signal-incompetent mice after the footpad inoculation of JEV, we measured the expression of several pro-inflammatory cytokines and chemokines in various peripheral tissues including pLN, spleen, mLN, iLN, and liver, as well as in CNS tissues including the SC and brain. As a result, IFN-I signal-incompetent mice showed persistent and highly increased levels of TNF-α, IL-6, CCL2, and CXCL2 mRNA expression in the various tissues tested at the periphery from 24 h pi, compared with IFN-I signal-competent mice (Fig. 5a). Similarly, these cytokines (TNF-α, IL-6) and chemokines (CCL2, CXCL2) were expressed in the CNS tissues of IFN-I signal-incompetent mice with higher levels than those of competent mice, even though the expression of pro-inflammatory cytokines in the CNS tissues were somewhat low and delayed compared with other peripheral tissues (Fig. 5b). In addition, we measured serum levels of vasoactive IL-6 and TNF-α proteins, which have been known to play an important role in inducing hemorrhagic fever and increasing vascular leakage [51, 54]. As expected, the levels of serum IL-6 and TNF-α were increased by 5- to 60-fold from 24 h pi, in IFN-I signal-incompetent mice compared with competent mice (Fig. 5c). The massive increase of multiple CC chemokines in sera of IFN-I signal-incompetent mice was also revealed, when we checked the levels of multiple CC chemokines in sera with CBA methods (Fig. 5d). This massive and uncontrolled production of multiple cytokines and chemokines in IFN-I signal-incompetent mice after JEV footpad inoculation was coupled with renal and hepatic injury and JEV Ags staining in tissues, and suggests a picture of sepsis due to “cytokine storm”.

### IFN-I signal is essentially required for quick innate IFN-I responses

**Fig. 6:**
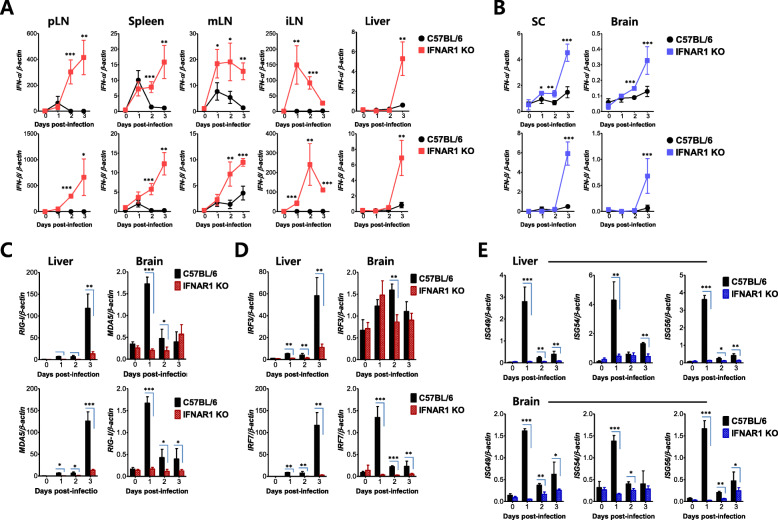
IFN-I signal-incompetent mice show impaired innate IFN-I responses. **a** and **b** The expression of IFN-I (IFN-α/β) mRNA in the peripheral and CNS tissues. **c** and **d** Impaired induction of RIG-I-like receptors (RLR) and IRF genes in the peripheral and CNS tissues of IFN-I signal-incompetent mice. **e** Impaired expression of IFN-stimulated genes (ISGs) in the peripheral and CNS tissues of IFN-I signal-incompetent mice. The mRNA levels of the indicated genes were determined by real-time qRT-PCR using total RNA extracted from popliteal LNs (pLN), spleen, mesenteric LNs (mLN), iliac LNs (iLN), liver, spinal cord (SC), and brain at the indicated days after JEV infection (5 × 10^6^ ffu/mouse) via the footpad route. Data in the graphs denote the mean ± SEM. Results are representative of one out of at least two individual experiments with four to five mice per group. Statistical significance *p < 0.05; **p < 0.01; ***p < 0.001 was assessed by an unpaired two-tailed Student’s *t* test

To better understand IFN-I innate responses in IFN-I signal-incompetent mice, we measured the expression of IFN-I (IFN-α/β) mRNA in various peripheral tissues including pLN, spleen, mLN, iLN, and liver after JEV footpad inoculation. Our data revealed that the expression of IFN-α/β mRNA in the peripheral tissues of IFN-I signal-incompetent mice increased 5 to 500 times at 48 and 72 h pi compared with the IFN-I signal-competent mice (Fig. 6a). Similarly, the CNS tissues (SC and brain) in IFN-I signal-incompetent mice displayed a higher expression of IFN-α/β mRNA 72 h after JEV footpad inoculation than IFN-I signal-competent mice (Fig. 6b). This implies that the ablation of the IFN-I signal did not induce blunted IFN-I production after JEV footpad inoculation and, rather, the magnitude of IFN-I (IFN-α/β) expression seemed to follow the degree of JEV replication because the replication of JEV was coupled to the expression levels of IFN-α/β. IFN-I (IFN-α/β) binds to a heterodimeric receptor (IFNAR1) and mediates downstream pleiotropic functions of the canonical JAK-STAT signaling pathway [23, 24]. This stimulation results in the induction of antiviral ISGs, induction of cell surface and cytosolic PRRs in antigen-presenting cells, and the regulation of cytokine and chemokine production [19]. Therefore, we measured the induction levels of antiviral ISGs to define IFN-I innate responses in greater detail. We specifically focused on the induction of PRRs (RIG-I [DDX1], MDA5 [IFITH1]), their transcription factors (IRF3, IRF7), and ISGs (ISG49 [IFIT3], ISG54 [IFIT2], ISG56 [IFIT1]). As a result, the expression of PRRs (RIG-I, MDA-5) was markedly elevated in IFN-I signal-competent mice compared with incompetent mice (Fig. 6c). Notably, RIG-I and MDA5 were induced by approximately 70-fold increased levels in the liver of IFN-I signal-competent mice at 3 dpi. Subsequently, IRF3 and IRF7 expression followed expression patterns of PRRs (RIG-I, MDA-5) in IFN-I signal-incompetent mice, except for IRF3 expression in brain tissue (Fig. 6d). The expression of ISGs (ISG49, ISG54, ISG56) was observed and peaked quickly at 1 dpi in IFN-I signal-competent mice, but IFN-I signal-incompetent mice showed no induction of ISG expression (Fig. 6e). Collectively, these results suggest that the impaired induction of ISG expression in the peripheral tissues, such as the liver, of IFN-I signal-incompetent mice at a very early stage was likely to contribute to the appearance of a hemorrhagic-like disease.

### Intrinsic IFN-I signaling in tissue-resident cells is critical to limiting hemorrhage-like diseases following JEV infection

**Fig. 7:**
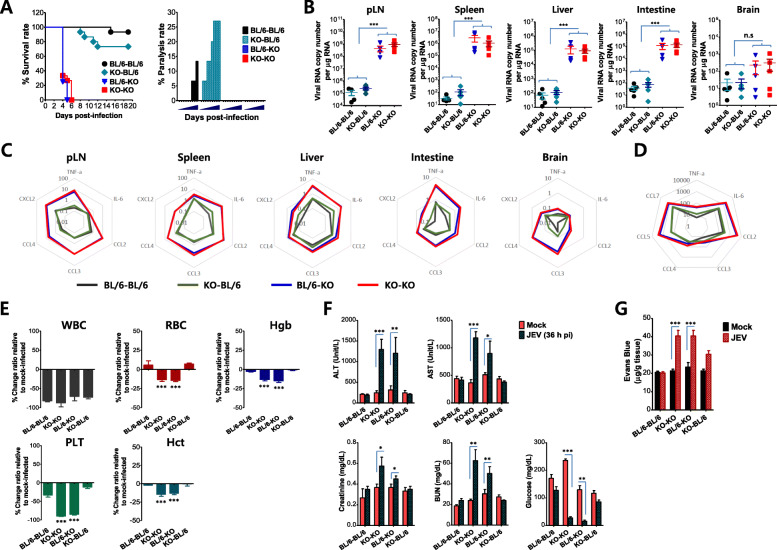
IFN-I signal in tissue-resident cells plays a crucial role in inducing CNS neuroinflammation through the peripheral restriction of viral dissemination. BM cells from wild-type C57BL/6 (BL/6) or IFNAR1 KO (KO) mice were grafted into lethally irradiated BL/6 or IFNAR1 KO recipient mice, which were infected with JEV (5 × 10^6^ ffu) via footpad route 5 weeks later. **a** Requirement of IFN-I signaling in tissue-resident cells for inducing CNS neuroinflammation. The proportion of surviving mice in each group was monitored daily for 20 days (left graph). Ratio of mice showing neurological disorder in inoculated recipients was recorded daily during JE progression (right graph). **b** Viral burden in the peripheral and CNS tissues of BM chimeric hosts. Viral burdens were assessed by real-time qRT-PCR using total RNA extracted from the peripheral and CNS tissues including popliteal LNs (pLN), spleen, liver, intestine, and brain 48 h pi. The viral RNA load was expressed by viral RNA copy number per microgram of total RNA (*n* = 4). Each symbol represents the level of an individual mouse; the horizontal line indicates the mean ± SEM of each group. **c** IFN-I signal deficiency in tissue-resident cells is required for high induction of inflammatory cytokine mRNA. The mRNA levels of the indicated cytokines and chemokines were determined by real-time qRT-PCR 48 h pi. Radar charts summarize the mean mRNA expression levels of the indicated cytokines in the indicated tissue with a log_10_ scale normalized to β-actin expression. **d** Indispensable role of IFN-I signal in tissue-resident cells in inducing “cytokine storm”. Serum levels of cytokines and CC chemokines were determined by cytokine ELISA (IL-6 and TNF-a) and cytokine bead array (CC chemokines) using sera collected from inoculated recipients 48 h pi. Radar chart shows the mean levels of the indicated cytokines with a log_10_ scale of cytokine level (pg/ml). **e** Complete blood count (CBC) data of BM chimeric hosts following JEV infection. The levels of white blood cells (WBC), red blood cells (RBC), hemoglobin (Hgb), hematocrit (Hct), and platelets (PLT) were determined using an automated hematology analyzer 48 h pi. Data are expressed by the change percentage compared to mock-infected recipients. **f** Liver and kidney functions of BM chimeric hosts following JEV infection. The injury of liver and kidney function was evaluated by the levels of ALT and AST for liver, and the levels of BUN, creatinine, and glucose for kidney using a clinical chemistry analyzer. **g** The amount of extravasated Evans blue dye in the intestine of JEV-infected BM chimeras. The amount of extravasated Evans blue dye was quantified by measuring the absorbance (620 nm) after homogenization and TCA-precipitation of the intestine tissue. Data in the graphs denote the mean ± SEM. Results are representative of one out of at least two individual experiments with four to five mice per group. Statistical significance *p < 0.05; **p < 0.01; ***p < 0.001 was assessed by an unpaired two-tailed Student’s *t* test

IFN-I signal-incompetent mice displayed hemorrhage-like disease after JEV inoculation at the distal site, and this altered disease appeared to be caused by impaired innate responses at a very early stage. However, the cell types that mainly contribute to the altered disease in IFN-I signal-incompetent mice remain to be defined. Therefore, we were interested in dissecting the contribution of tissue-resident cells and HSC-derived leukocytes in this process. To achieve this, we used a BM chimeric model established using IFN-I signal-competent (BL/6) and -incompetent (IFNAR1 KO) mice. Strikingly contrasting results were revealed in our BM chimeric experiments. IFNAR1 KO recipients of BL/6 WT BM cells (BL/6–KO chimera) or IFNAR1 KO BM cells (KO–KO chimera) showed the same susceptibility to JEV footpad inoculation, whereas BL/6 WT recipients of IFNAR1 KO BM cells (KO–BL/6 chimera) or BL/6 WT BM cells (BL/6–BL/6 chimera) displayed almost the same resistance to JEV infection (Fig. 7a, *left graph*). Furthermore, the BL/6–KO chimera died without neurological disorders like the KO–KO chimera, but the KO–BL/6 chimera succumbed to neurological disorders that showed higher frequencies than the BL/6–BL/6 chimera (Fig. 7a, *right graph*). To better understand this contrasting result in the BM chimeric experiments, we checked the viral load in various peripheral tissues including pLN, spleen, liver, and intestine, as well as in CNS tissues such as the brain. In support, similar to KO–KO chimera, BL/6–KO chimera showed markedly increased loads of JEV in various peripheral tissues 48 h pi, as compared with both KO–BL/6 and BL/6–BL/6 chimeras (Fig. 7b). No significant differences in JEV loads in peripheral tissues were observed between KO–KO and BL/6–KO chimeras. However, both BL/6–KO and KO–KO chimeras showed no significantly increased loads of JEV in CNS tissues, such as the brain, compared with those of KO–BL/6 and BL/6–BL/6 chimeras, which indicates that tissue-resident cells play an important role in disseminating JEV into the peripheral lymphoid and non-lymphoid tissues after JEV footpad inoculation.

Next, we checked the expression of pro-inflammatory cytokines and chemokines in the peripheral lymphoid and non-lymphoid tissues as well as in CNS tissues, to further characterize the disease induced in BM chimeric models after JEV footpad inoculation. BL/6–KO chimera showed highly enhanced levels of pro-inflammatory cytokines (IL-6, TNF-α) and chemokines (CCL2, CCL3, CCL4, CXCL2) in the peripheral tissues, as shown in KO–KO chimera (Fig. 7c). However, the expression of pro-inflammatory cytokines and chemokines in the brain was at almost the same low level in the four BM chimeric models, except for certain chemokines such as CCL2, CCL3, and CCL4. Similarly, pro-inflammatory cytokine and chemokine proteins in the sera of the BL/6–KO chimera were detected at markedly higher levels compared with KO–BL/6 and BL/6–BL/6 chimeras (Fig. 7d), which indicates that the BL/6–KO chimera displayed a “cytokine storm” after JEV footpad inoculation, as occurred in IFN-I signal-incompetent mice. Because the IFN-I signal-incompetent mice showed hemorrhage-like disease through thrombocytopenia and altered hematocrit after JEV footpad inoculation, we checked blood chemistries of the four BM chimeric models. The marked reductions in the values of RBC, hemoglobin, platelet, and hematocrit were observed in both BL/6–KO and KO–KO BM chimeras, as compared with BL/6–BL/6 and KO–BL/6 chimeras (Fig. 7e). Notably, BL/6–KO BM chimera have severe thrombocytopenia, thereby resulting in a reduction of hematocrit value, as shown in IFN-I signal-incompetent mice after JEV footpad inoculation. Furthermore, to evaluate whether the BL/6–KO BM chimera had the peripheral organ injury found in IFN-I signal-incompetent mice, we checked liver and kidney functions in the four BM chimeras after JEV footpad inoculation. As expected, the BL/6–KO BM chimera had highly enhanced values of ALT and AST, which indicates that the BL/6–KO BM chimera has impaired liver function after JEV infection (Fig. 7f). Similarly, increased creatinine and BUN values in the BL/6–KO BM chimera indicated impaired kidney functions and displayed hypoglycemia, as shown in IFN-I signal-incompetent mice after JEV infection. Finally, we assessed the integrity of the vascular endothelial barrier using the Evan blue dye extravasation method. Enhanced vascular leakages confirmed that the BL/6–KO BM chimera had the hemorrhage-like disease shown in IFN-I signal-incompetent mice after JEV footpad inoculation (Fig. 7g). Summarizing all the results derived from the BM chimeric experiments, our data suggest that IFN-I signaling in tissue-resident cells plays a critical role in restricting hemorrhage-like disease after JEV inoculation at the distal site.

### IFN-I signaling attenuates JEV replication in hepatocytes, enterocytes, and neuron cells

**Fig. 8:**
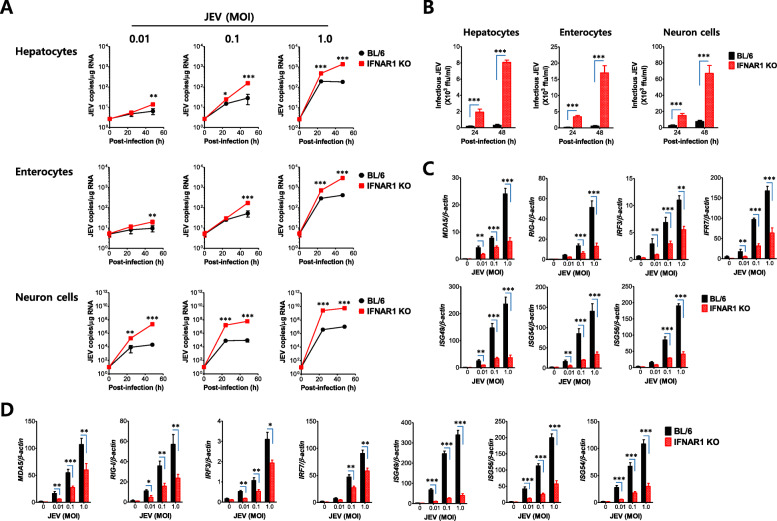
IFN-I signal is required to attenuate JEV replication in hepatocytes, enterocytes, and neuron cells. Primary hepatocytes, enterocytes, and cortical neuron cells generated from IFN-I signal-competent and -incompetent mice were infected with JEV at the indicated MOI (0.01, 0.1, and 1.0). **a** The replication of JEV RNA genome in hepatocytes, enterocytes, and neuron cells. JEV replication was assessed by detecting viral RNA levels with real-time qRT-PCR at the indicated time points pi. Viral RNA levels were expressed by JEV RNA copy number per microgram of total RNA. **b** The production of infectious JEV in hepatocytes, enterocytes, and neuron cells. The levels of infectious JEV in culture media were determined by focus-forming assay following JEV infection (1.0 MOI). **c** and **d** Impaired induction of RIG-I-like receptors (RLRs), IRFs, and IFN-stimulated genes (ISGs) in IFN-I signal-deficient hepatocytes and neuron cells. The mRNA levels of the indicated genes were determined by real-time qRT-PCR using total RNA extracted from primary hepatocytes (**c)** and cortical neuron cells (**d)** 48 h following JEV infection with different doses (0.01, 0.1, and 1.0 MOI). Data in the graphs denote the mean ± SEM derived from quadruplicated wells. Results are representative of one out of at least two individual experiments. Statistical significance *p < 0.05; **p < 0.01; ***p < 0.001 was assessed by an unpaired two-tailed Student’s *t* test

The intrinsic role of IFN-I signal in tissue-resident cells, rather than HSC-derived cells, in suppressing viral dissemination and hemorrhage-like disease after JEV footpad inoculation was proposed. Therefore, we were interested in the effect of IFN-I signaling on JEV replication in tissue-resident cells, in order to further define cellular factors for viral dissemination and hemorrhage-like disease in IFN-I signal-incompetent mice. To achieve this, we prepared primary hepatocytes, enterocytes, and cortical neuron cells from IFN-I signal-competent and -incompetent mice, and then infected those primary cells to check viral permissiveness. JEV was observed to replicate 10- to 100-fold more rapidly in primary hepatocytes, enterocytes, and neuron cells derived from IFN-I signal-incompetent mice compared with IFN-I signal-competent mice (Fig. 8a). Notably, JEV replicated much more quickly at 10^5^- to 10^8^-fold increased levels in primary neuron cells, compared with other primary cells including hepatocytes and enterocytes. This result indicates that IFN-I signaling is very important in regulating JEV replication in tissue-resident cells including hepatocytes, enterocytes, and neuron cells, and demonstrates that primary neuron cells are inherently and very permissive to JEV replication, compared with other tissue-resident cells such as hepatocytes and enterocytes. In support, infectious JEV was recovered with highly increased levels in primary hepatocytes, enterocytes, and neuron cells derived from IFN-I signal-incompetent mice compared with competent mice, and primary neuron cells produced infectious JEV with higher levels than other primary cells (Fig. 8b). To better understand the rapid replication of JEV in tissue-resident cells derived from IFN-I signal-incompetent mice, we examined the induction of antiviral ISGs in primary hepatocytes and neuron cells after JEV infection. As expected, primary hepatocytes derived from IFN-I signal-incompetent mice showed highly impaired induction of antiviral ISGs including MDA5, RIG-I, IRF3, and IRF7, as well as ISG49, ISG54, and ISG56, depending on the infectious dose of JEV (Fig. 8c). Primary neuron cells derived from IFN-I signal-incompetent mice also showed reduced induction of antiviral ISGs following JEV infection (Fig. 8d). Collectively, these results suggest that tissue-resident cells derived from IFN-I signal-incompetent mice are very permissive to JEV replication, due to impaired induction of antiviral ISGs. In addition, our data imply that neuron cells are inherently and highly vulnerable to JEV infection compared with other tissue-resident cells.

### Early and higher infection of circulating CD11b^+^Ly-6C^+^ monocytes at the inoculation site is a prerequisite to JEV dissemination

**Fig. 9:**
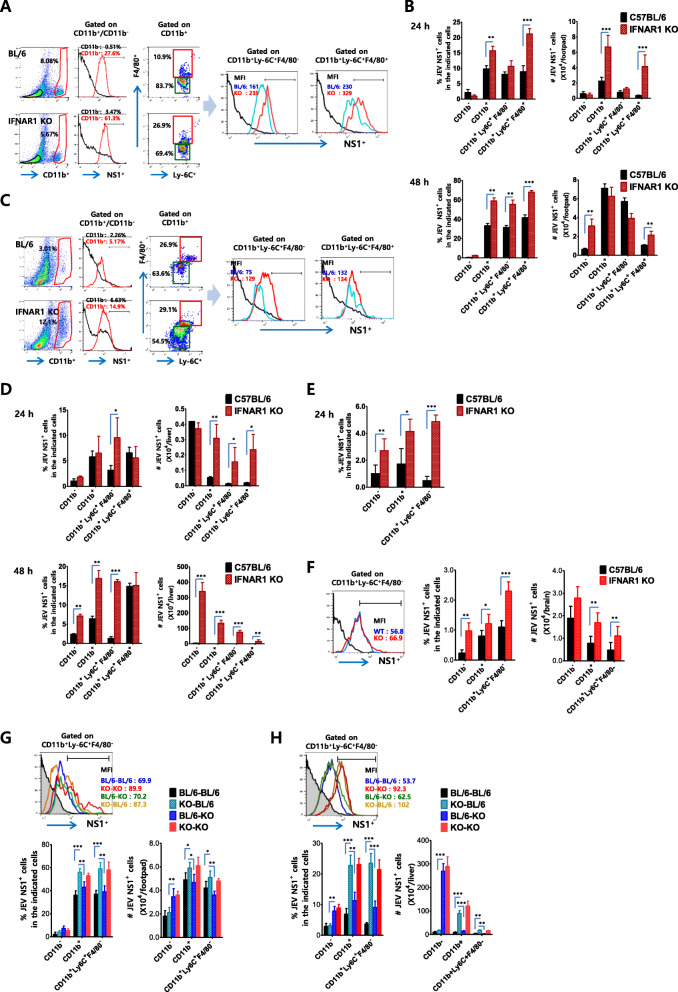
Early and higher infection of circulating CD11b^+^Ly-6C^+^ monocytes in the infection site of IFN-I signal-incompetent mice is associated with quick viral dissemination. **a** Prerequisite infection of CD11b^+^Ly-6C^+^ monocytes in primary inoculation site of JEV. Cells were prepared from peripheral tissues (footpad) for primary inoculation site of JEV (5 × 10^6^ ffu/mouse) by collagenase digestion 48 h pi. Subpopulations of JEV NS1 protein-positive leukocytes were detected using flow cytometric analysis after staining cells with myeloid and monocyte/macrophage markers (CD11b, Ly-6C, F4/80) combined with Mab against JEV NS1 protein. **b** Earlier, higher frequency and number of JEV Ag-positive CD11b^+^Ly-6C^+^ monocytes in primary inoculation sites of IFN-I signal-incompetent mice. JEV NS1 protein-positive CD11b^+^ subpopulations were assessed by flow cytometric analysis using footpad-derived cells stained with CD11b, Ly-6C, and F4/80 combined with JEV NS1 antibody 24 and 48 h pi. **c** Detection of JEV-infected CD11b^+^Ly-6C^+^ monocytes in the liver following JEV infection. Cells were prepared from liver tissues by collagenase perfusion digestion 48 h pi and used for the analysis of JEV NS1-positive cells. **d** Enhanced JEV infection of liver CD11b^+^Ly-6C^+^ monocytes in IFN-I signal-incompetent mice. JEV NS1 protein-positive CD11b^+^ subpopulations were assessed by flow cytometric analysis 24 and 48 h pi. **e** Enhanced infection of IFN-I signal-deficient CD11b^+^Ly-6C^+^ monocytes in blood. Collected PBL was stained with CD11b, Ly-6C, and F4/80 combined with JEV NS1 antibody 24 h pi. JEV NS1-positive cells were assessed by flow cytometric analysis. **f** Enhanced CNS infiltration of JEV Ag-positive CD11b^+^Ly-6C^+^ monocytes in IFN-I signal-incompetent mice. Infiltrated leukocytes prepared from brain of IFN-I signal-competent and -incompetent mice were used to analyze JEV Ag-positive CD11b^+^Ly-6C^+^ monocytes with flow cytometry 48 h pi. **g** and **h** Involvement of CD11b^+^Ly-6C^+^ monocytes in quick viral dissemination in IFN-I signal-incompetent mice using BM chimeric models. Infiltrated leukocytes prepared from footpad and liver of BM chimeric hosts were used for the detection of JEV Ag-positive CD11b^+^Ly-6C^+^ monocytes 48 h pi. Data in bar graphs denote the mean ± SEM. Results are representative of one out of at least two individual experiments with four to five mice per group. Statistical significance *p < 0.05; **p < 0.01; ***p < 0.001 was assessed by an unpaired two-tailed Student’s *t* test

Tissue-resident cells that are deficient in IFN-I signaling showed high permissiveness to JEV infection and played a dominant role in inducing hemorrhage-like disease in IFN-I signal-incompetent mice after JEV footpad inoculation, due to enhanced JEV dissemination into peripheral organ tissues. Here, we were interested in identifying cellular factors that carry the virus throughout the body after JEV inoculation at the distal peripheral sites. To this end, we screened various cell types that could be infected in footpad tissue at an early phase (24 and 48 h pi) after JEV inoculation. Eventually, we found that CD11b^+^ myeloid cells were preferentially infected 48 h after JEV infection in the footpad tissues of IFN-I signal-incompetent mice compared with competent mice (Fig. 9a). Furthermore, our analysis revealed that CD11b^+^Ly-6C^+^ monocytes were highly infected in footpad CD11b^+^ myeloid cell population derived from IFN-I signal-incompetent mice. Notably, CD11b^+^Ly-6C^+^ inflammatory macrophages that show mature macrophage phenotype (F4/80-positive) in the footpad of IFN-I signal-incompetent mice were infected with highly increased levels, as shown by the high frequency of CD11b^+^Ly-6C^+^F4/80^+^ inflammatory macrophages with an NS1 JEV Ag-positive response. Similarly, the footpad derived from IFN-I signal-incompetent mice contained highly increased number of NS1-positive CD11b^+^Ly-6C^+^F4/80^+^ inflammatory macrophages compared with competent mice, and the footpad of IFN-I signal-incompetent mice contained higher number of NS1-postive CD11b^+^Ly-6C^+^F4/80^+^ inflammatory macrophages at 24 h than 48 h (Fig. 9b), which indicates that JEV-infected CD11b^+^Ly-6C^+^F4/80^+^ inflammatory macrophages were detected earlier and higher in the footpad of IFN-I signal-incompetent mice than IFN-I signal-competent mice. Subsequently, we examined JEV-infected CD11b^+^Ly-6C^+^ monocytes in peripheral organ tissues such as the liver after JEV inoculation at the distal footpad tissue. CD11b^+^Ly-6C^+^ monocytes derived from the liver of IFN-I signal-incompetent mice were infected at higher levels compared to competent mice (Fig. 9c). However, unlike the footpad tissue, liver CD11b^+^Ly-6C^+^F4/80^+^ inflammatory macrophages matured from monocytes had a comparable infection rate in both IFN-I signal-competent and -incompetent mice. In support, JEV-infected CD11b^+^Ly-6C^+^ monocytes were detected at higher levels in the liver of IFN-I signal-incompetent mice compared with competent mice 24 h after JEV footpad inoculation, while CD11b-negative non-myeloid cells showing NS1 JEV Ag-positive response were detected at comparable levels (Fig. 9d). The next day (48 h pi), liver tissue derived from IFN-I signal-incompetent mice contained at high levels of JEV-infected CD11b^+^Ly-6C^+^ monocytes as well as CD11b-negative non-myeloid cells compared with competent mice. These results indicate that CD11b^+^Ly-6C^+^ monocytes and their matured macrophages could be preferentially infected in footpad tissue at an early stage in JEV infection, after which JEV-infected monocytes appear to carry the virus to peripheral organs, such as the liver. CD11b^+^Ly-6C^+^ monocytes that are recruited into inflamed tissues such as the footpad differentiate into macrophages, and some monocytes escape the inflamed tissues and enter the blood, thereby spreading the virus to entire peripheral organs [55–57]. In support of this, when we analyzed JEV-infected CD11b^+^Ly-6C^+^ monocytes in the blood, our results revealed that blood CD11b^+^Ly-6C^+^ monocytes were infected with highly increased levels in IFN-I signal-incompetent mice compared with competent mice 24 h after JEV footpad inoculation (Fig. 9e). In addition, we evaluated the frequency and number of JEV-infected CD11b^+^Ly-6C^+^ monocytes infiltrated into the CNS after JEV inoculation at footpad tissues, because IFN-I signal-incompetent mice showed modestly increased levels of JEV RNA burden and infectious virus in the brain 48 h pi, compared with IFN-I signal-competent mice. As expected, CD11b^+^Ly-6C^+^ monocytes derived from the CNS of IFN-I signal-incompetent mice showed higher levels of JEV Ag (NS1) expression than those of IFN-I signal-competent mice (Fig. 9f). IFN-I signal-incompetent mice contained increased frequency and number of JEV NS1-positive CD11b^+^ myeloid cells and CD11b^+^Ly-6C^+^ monocytes in the brain. However, JEV NS1-positive CD11b^-^ tissue-resident cells in the brain of IFN-I signal-incompetent mice were detected with modestly but not significantly increased levels compared with IFN-I signal-competent mice. This result may support that IFN-I signal-incompetent mice showed modestly but not significantly increased levels of JEV burden in the CNS at 48 h pi, compared with IFN-I signal-competent mice. In order to further characterize the role of CD11b^+^Ly-6C^+^ monocytes in quick viral dissemination into the peripheral tissue-resident cells of IFN-I signal-incompetent mice, we used BM chimeric hosts infected with JEV via footpad route, and determined the frequency and number of JEV NS1-positive CD11b^+^Ly-6C^+^ monocytes in the footpad and liver of BM chimeric hosts. In support, our data revealed that IFN-I signal-deficient CD11b^+^Ly-6C^+^ monocytes showed higher expression of JEV NS1 protein than IFN-I signal-competent monocytes (Fig. 9g, h). Here, one interesting data was that CD11b-negative tissue-resident cells in footpad and liver of IFN-I signal-incompetent recipients of BM cells derived from IFN-I signal-competent mice (BL/6–KO BM chimeric host) showed higher expression of JEV NS1 protein compared to KO–BL/6 BM chimeric host. This result indicates that IFN-I signal-incompetent tissue-resident cells are permissive to inoculation of JEV delivered by CD11b^+^Ly-6C^+^ monocytes.

**Fig. 10:**
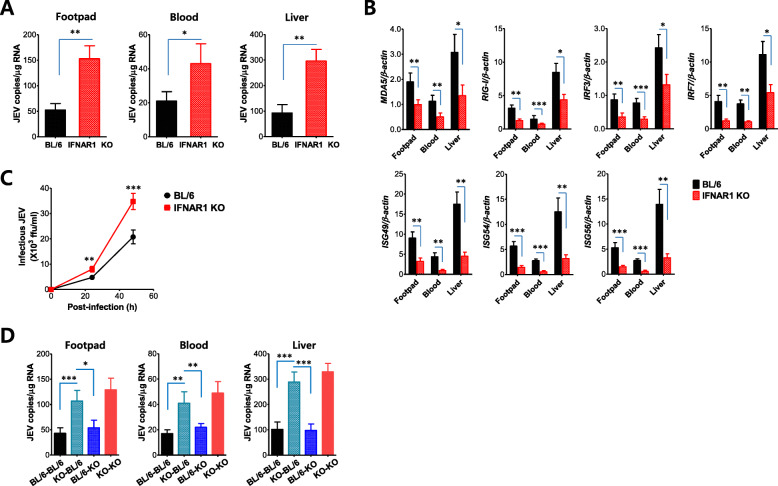
Circulating IFN-I signal-deficient CD11b^+^Ly-6C^+^ monocytes are highly permissive to JEV replication. Cells were prepared from the primary inoculation site (footpad), blood, and liver of IFN-I signal-competent and -incompetent mice 48 h after JEV infection (5 × 10^6^ ffu/mouse) via the footpad route, and used to purify CD11b^+^Ly-6C^+^ monocytes using a FACS Aria sorter. **a** Viral burden of circulating CD11b^+^Ly-6C^+^ monocytes in footpad, blood, and liver. Viral RNA load was determined by real-time qRT-PCR using total RNA extracted from sorted CD11b^+^Ly-6C^+^ monocytes. Viral RNA levels were expressed by JEV RNA copy number per microgram of total RNA. **b** Induction of RIG-I-like receptors (RLRs), IRFs, and IFN-stimulated genes (ISGs) in CD11b^+^Ly-6C^+^ monocytes derived from the footpad, blood, and liver. The mRNA levels of the indicated genes were determined by real-time qRT-PCR using total RNA extracted from sorted CD11b^+^Ly-6C^+^ monocytes. **c** Enhanced infection of CD11b^+^Ly-6C^+^ monocytes derived from blood of IFN-I signal-incompetent mice. CD11b^+^Ly-6C^+^ monocytes were purified from PBL collected from uninfected IFN-I signal-competent and -incompetent mice, and infected with JEV (1.0 MOI) in the presence of GM-CSF (10 ng/ml). Infectious JEV in culture media was determined by a focus-forming assay. **d** Enhanced JEV burden of IFN-I signal-incompetent CD11b^+^Ly-6C^+^ monocytes in BM chimeric hosts. CD11b^+^Ly-6C^+^ monocytes were purified from the footpad, blood, and liver of BM chimeric hosts 48 h pi, and used for determining JEV burden with real-time qRT-PCR. Data in bar graphs denote the mean ± SEM. Results are representative of one out of at least two individual experiments with four to five mice per group. Statistical significance *p < 0.05; **p < 0.01; ***p < 0.001 was assessed by an unpaired two-tailed Student’s *t* test

To better understand the preferential infection of JEV in CD11b^+^Ly-6C^+^ monocytes, we measured JEV loads in CD11b^+^Ly-6C^+^ monocytes purified from the footpad, blood, and liver. As expected, CD11b^+^Ly-6C^+^ monocytes purified from the footpad, blood, and liver of IFN-I signal-incompetent mice contained 2- to 3-fold increased levels of JEV RNA compared with IFN-I signal-competent mice (Fig. 10a). In support, CD11b^+^Ly-6C^+^ monocytes purified from the footpad, blood, and the liver of IFN-I signal-incompetent mice showed impaired induction of antiviral ISGs (MDA5, RIG-I, IRF3, IRF7, ISG49, ISG54, ISG56) compared to IFN-I signal-competent mice (Fig. 10b). Also, in order to examine infectious JEV production in CD11b^+^Ly-6C^+^ monocytes, we infected CD11b^+^Ly-6C^+^ monocytes purified from the blood of IFN-I signal-competent and -incompetent mice, and determined the production of infectious JEV. Our data revealed that CD11b^+^Ly-6C^+^ monocytes purified from IFN-I signal-incompetent mice produced infectious JEV with higher levels than IFN-I signal-competent mice (Fig. 10c). Finally, we measured JEV loads in CD11b^+^Ly-6C^+^ monocytes purified from the footpad, blood, and liver of BM chimeric hosts 48 h after JEV inoculation at footpad tissues. As expected, IFN-I signal-deficient CD11b^+^Ly-6C^+^ monocytes derived from KO–BL/6 and KO–KO BM chimeric hosts contained higher JEV RNA burden than BL/6–KO and BL/6–BL/6 BM chimeric hosts (Fig. 10d). Taken together, these results suggest that preferential infection of CD11b^+^Ly-6C^+^ monocytes in the footpad tissue of IFN-I signal-incompetent mice is required for quick viral dissemination into the entire body. At an early stage after JEV footpad inoculation, JEV-infected CD11b^+^Ly-6C^+^ monocytes in the footpad are likely to carry the virus through the blood into peripheral tissues.

## Discussion

IFN-I signal has been well known to play an important role in controlling viral infection. In particular, the critical contribution of IFN-I signal and transcription factors (IRF3, IRF5, IRF7, IPS-1, etc.) for IFN-I production to the control of in vivo flavivirus infection mostly focused on WNV has been described [20–22]. Although IFN-I signaling is thought to play an important role in providing protective immunity to JEV infection, there has been no research on the role of IFN-I signaling in CNS neuroinflammation using a practical in vivo JEV infection model. In addition, IFN-I signal-dependent cellular factors responsible for CNS neuroinflammation after JEV infection are still ambiguous. Our results propose a mechanistic model for how CD11b^+^Ly-6C^+^ monocytes are involved in the tissue tropism of JEV to the CNS, depending on the IFN-I signal. In the present study, we found that IFN-I signal-competent mice died by a prolonged and neurological illness following JEV inoculation in distal tissue, whereas IFN-I signal-incompetent mice died without any neurological signs. Rather, IFN-I signal-incompetent mice had a hemorrhage-like disease and quick viral dissemination through the entire body, as evidenced by blood cell analysis, parameters for liver and kidney functions, and increased vascular leakage. The massive and uncontrolled production of pro-inflammatory cytokines and chemokines in sera of IFN-I signal-incompetent mice was closely associated with the hemorrhage-like disease. Using a BM chimeric model, intrinsic IFN-I signaling in tissue-resident cells was found to play a major role in inducing hemorrhage-like disease, as IFN-I signal-incompetent recipients for BM cells derived from IFN-I signal-competent mice showed enhanced JEV dissemination, uncontrolled cytokine production, and increased vascular leakage. IFN-I signal-deficient hepatocytes and enterocytes were permissive to JEV replication with impaired induction of antiviral ISGs, and neuron cells derived from IFN-I signal-competent and -incompetent mice showed inherent and high vulnerability to JEV replication. This indicated that neurons could be a major cell for JEV replication once it has invaded the CNS. Finally, we showed that circulating CD11b^+^Ly-6C^+^ monocytes infiltrated into distal tissue inoculated with JEV played a role in the quick dissemination of JEV to peripheral organs of IFN-I signal-incompetent mice at an early stage.

CNS immune privilege is maintained by multiple factors, including its isolation from the peripheral immune system by the blood–brain barrier (BBB), lack of draining lymphatics, and the apparent immunocompetence of microglia [58]. The infiltration of CD11b^+^Ly-6C^+^ monocytes into CNS immune privilege is a hallmark of CNS neuroinflammation caused by neurotrophic viruses, such as JEV and WNV [59–61]. During the course of inflammation, CD11b^+^Ly-6C^+^ monocytes are specifically recruited to inflammatory sites in various conditions from the BM via blood, depending on the CCR2–CCL2 axis [55, 62]. CD11b^+^Ly-6C^+^ monocytes recruited into inflamed tissue, such as the footpad, inoculated with JEV differentiate into CD11b^+^Ly-6C^+^F4/80^+^ inflammatory macrophages, and some monocytes leave the inflamed tissues and enter the blood, then move to other organs [55–57]. In addition, CD11b^+^Ly-6C^+^F4/80^+^ inflammatory macrophages differentiated from CD11b^+^Ly-6C^+^ monocytes migrate draining lymph nodes and/or activate effector T cells into inflamed tissues [63, 64]. The migration and differentiation of CD11b^+^Ly-6C^+^ monocytes during the inflammatory process are very complicated, and there is a limit to subdividing and understanding their migration and differentiation. Likewise, even though the possibility of JEV traveling through the plasma of blood and the spread of virus into lymph nodes has not been completely ruled out, our data suggest that JEV spreads to other peripheral organs via CD11b^+^Ly-6C^+^ monocytes in the absence of IFN-I signaling. After screening various cell types positive for JEV NS1 Ag, we found that CD11b^+^Ly-6C^+^ monocytes and their matured CD11b^+^Ly-6C^+^F4/80^+^ macrophages were preferentially positive for JEV NS1 Ag in the JEV inoculation site (footpad) in the early stage (1 dpi) of infection. The delivery of inoculated JEV from the primary infection site to other peripheral organs is believed to be involved in the later hemorrhage-like disease in IFN-I signal-incompetent mice. In support of this suggestion, our data revealed that CD11b^+^Ly-6C^+^ monocytes were positive for JEV NS1 Ag in the liver at high levels on the second day (48 h) after JEV footpad infection, whereas the number of JEV Ag-positive CD11b^+^Ly-6C^+^ monocytes in the footpad decreased. It is thought that CD11b^+^Ly-6C^+^ monocytes in the footpad on the first day migrated to the liver on the second day carrying JEV in the bloodstream. This notion is strengthened by the results that IFN-I signal-deficient CD11b^+^Ly-6C^+^ monocytes derived from KO–BL/6 BM chimeric host displayed higher expression of JEV NS1 protein than BL/6–KO BM chimeric host, whereas CD11b-negative tissue-resident cells in BL/6–KO BM chimeric host showed higher expression of JEV NS1 protein than KO–BL/6 BM chimeric host. In addition, this speculation is partially supported by the result showing that CD11b^+^Ly-6C^+^ monocytes derived from the footpad of IFN-I signal-incompetent mice contained higher levels of JEV RNA than those of competent mice. Here, it is unclear whether CD11b^+^Ly-6C^+^ monocytes egressed from the footpad and moved back to the BM. Low levels of JEV NS1-positive CD11b^+^Ly-6C^+^ monocytes in the BM were comparable in both IFN-I signal-competent and -incompetent mice (Data not shown). Therefore, it is presumed that CD11b^+^Ly-6C^+^ monocytes leaving the footpad migrated to the peripheral organs such as the liver, and not the BM.

**Fig. 11:**
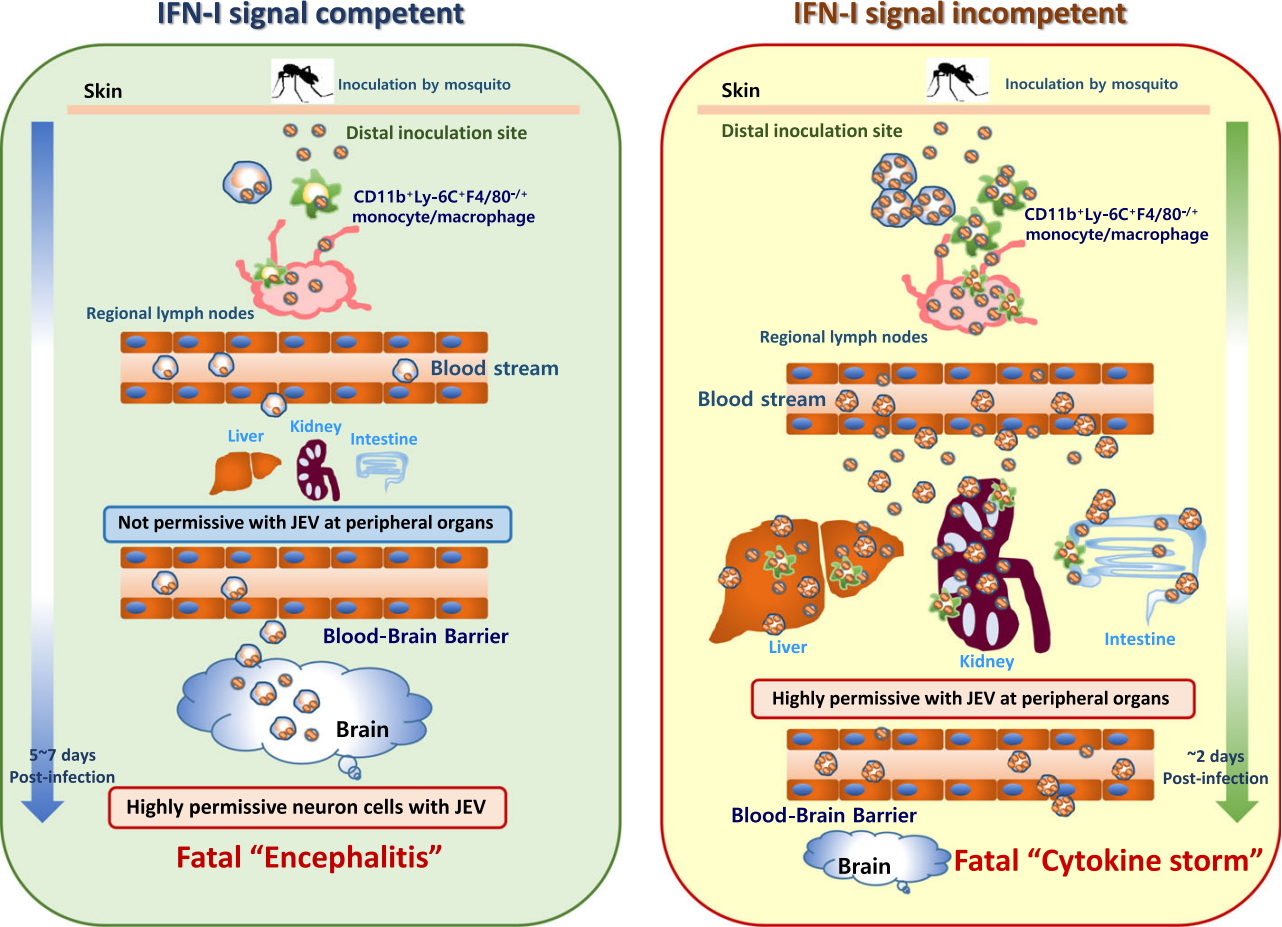
Proposed model for IFN-I role in inducing CNS neuroinflammation through the peripheral restriction of JEV dissemination. In IFN-I signal-competent hosts, JEV first infects locally infiltrated CD11b^+^Ly-6C^+^ monocytes and their matured CD11b^+^Ly-6C^+^F4/80^+^ macrophages in primary inoculation tissues (footpad) and then in local LNs (popliteal LNs). Virus produced in these sites gains entry into the bloodstream via CD11b^+^Ly-6C^+^ monocytes, causing primary viremia. Subsequently, the virus is carried to larger organs such as the liver, kidney, intestine, and so forth, but tissue-resident cells in those organs are not permissive to virus replication. Later (5–7 dpi), CD11b^+^Ly-6C^+^ monocytes infected with JEV gain access to the CNS, which inoculate virus to very permissive neuron cells, thereby resulting in encephalitis. In contrast, JEV is provided with higher levels in IFN-I signal-incompetent mice after infecting infiltrated CD11b^+^Ly-6C^+^ monocytes and their matured CD11b^+^Ly-6C^+^F4/80^+^ macrophages in primary inoculation sites and then in local LNs. Virus appears in the bloodstream through circulating CD11b^+^Ly-6C^+^ monocytes, and subsequently infects permissive tissue-resident cells (hepatocytes, enterocytes, etc.) in the liver, kidney, and intestine, and so forth. Infection of various tissue-resident cells in larger organs with JEV brings about a fatal “cytokine storm” in IFN-I signal-incompetent hosts within 2 dpi, thereby inducing multiple organ failure rather than encephalitis

The more interesting finding was that tissue-resident cells were the main players in causing the alteration of tissue tropism in the absence of IFN-I signaling, and thereby resulting in the induction of a hemorrhage-like disease. The individual cell types are considered to respond differentially to signaling by the binding of IFN-I to IFNAR1 because individual cell types have shown overlapping yet distinct transcriptional programs [37, 38]. Notably, targeting of the viral life cycle at an early step by multiple and novel antiviral ISGs indicated that host cells use several strategies to engender a single outcome such as translational inhibition [38]. In addition, two types of neuron cells derived from distinct brain regions were found to have unique innate immune signatures that may contribute to their relative permissiveness to viral infection [37]. Similarly, hepatocytes were observed to display overlapping but different levels of ISGs from neuron cells with JEV infection, because antiviral ISGs including IRF3, IRF7, ISG49, and ISG54 were expressed in hepatocytes at higher levels than in neuron cells. IFN-I signal-competent hepatocytes and enterocytes were markedly resistant to JEV infection, and neurons showed inherent vulnerability to JEV infection, regardless of whether IFN-I signaling was present. However, IFN-I signal-deficient hepatocytes and enterocytes produced 10-fold increased levels of infectious JEV compared with IFN-I signal-competent hepatocytes and enterocytes. This implies that tissue-resident cells such as hepatocytes and enterocytes have no permissiveness to JEV infection because of inherent and antiviral properties in IFN-I signal-competent mice, but those tissue-resident cells may be productive for infectious JEV in IFN-I signal-incompetent mice. Conceivably, it is presumed that tissue-resident cells in the liver and kidney play a role in inducing hemorrhage-like disease in IFN-I signal-incompetent mice through receiving JEV transported by CD11b^+^Ly-6C^+^ monocytes from footpad inoculation. Based on these speculations, we propose mechanistic models for how IFN-I signaling can control the CNS-restricted tissue tropism of JEV (Fig. 11). JEV first infects locally infiltrated CD11b^+^Ly-6C^+^ monocytes and their matured CD11b^+^Ly-6C^+^F4/80^+^ macrophages in primary inoculation tissues (footpad) and then in local LNs (pLNs) in IFN-I signal-competent hosts. Virus produced in these sites gain entry into the bloodstream via CD11b^+^Ly-6C^+^ monocytes, causing primary viremia. JEV is transported to larger organs such as the liver, kidney, intestine, and so forth, but tissue-resident cells in those organs are not permissive to JEV replication. Later (5–7 dpi), CD11b^+^Ly-6C^+^ monocytes infected with JEV gain access to the CNS under certain circumstances, and very permissive neuron cells are subsequently inoculated with virus, thereby resulting in encephalitis. In contrast, JEV is provided at higher levels in IFN-I signal-incompetent mice after infecting infiltrated CD11b^+^Ly-6C^+^ monocytes and their matured CD11b^+^Ly-6C^+^F4/80^+^ macrophages in primary inoculation sites and then in local LNs. Virus appears in the bloodstream through circulating CD11b^+^Ly-6C^+^ monocytes, which subsequently infects permissive tissue-resident cells (hepatocytes, enterocytes, etc.) in the liver, kidney, and intestine of IFN-I signal-incompetent hosts. Infection of various tissue-resident cells in larger organs with JEV brings about a fatal “cytokine storm” in IFN-I signal-incompetent hosts within 2 dpi, thereby inducing multiple organ failure rather than encephalitis.

A “cytokine storm” is a serological phenomenon in which an excessive amount of cytokines is produced in sera following infection by gram-negative bacteria or pathogenic influenza A and coronaviruses such as SARS-CoV2 [65–70]. Excessive cytokine production with the alteration of vascular permeability has also been described for flaviviruses in the context of severe DenV infection in human or highly immunocompromised mice [71, 72]. No blunted production of pro-inflammatory cytokines and IFN-I (IFN-αβ) was observed in IFN-I signal-incompetent mice following footpad inoculation of JEV. Even IFN-I signal-incompetent mice produced markedly increased production of pro-inflammatory cytokines and chemokines in sera, as well as IFN-I (IFN-α/β) expression in peripheral tissues. Although the susceptibility of IFN-I signal-incompetent mice to JEV infection is related to the higher levels of JEV replication in peripheral tissues, the contribution of the cytokine storm was not assessed. It can be conceived that excessive production of cytokines and chemokines in the sera of IFN-I signal-incompetent mice is achieved through the stimulation of PRRs by increasing JEV replication. However, on the second day after JEV footpad inoculation, immunohistochemistry using JEV Ag staining revealed less JEV RNA replication than anticipated in the liver and lymphoid tissues. This makes us consider injury to the peripheral organs such as the liver and kidney by pro-inflammatory cytokines released in the sera of IFN-I signal-incompetent mice. Although multifocal necrosis was found in peripheral organs such as the liver, vasoactive cytokines including IL-6 and TNF-α affect blood vessel permeability and vascular tone [51, 54], which suggests that excessive production of IL-6 and TNF-α increases vascular leakage in IFN-I signal-incompetent mice, one of the hallmarks of hemorrhage diseases [51, 54]. Markedly decreased expression of TJs in IFN-I signal-incompetent mice may support this notion. Additionally, enhanced production of chemokines in sera of IFN-I signal-incompetent mice may cause uncontrolled infiltration of blood leukocytes into the parenchymal tissues of peripheral organs, resulting in necrosis of parenchymal tissues, as evidenced by increased expression of adhesion molecules ICAM-1 and JAM in IFN-I signal-incompetent mice. IFN-I signal-incompetent mice developed marked elevations in serum liver enzymes (ALT and AST) and profound hypoglycemia, which is consistent with liver and kidney injury. Similarly, marked changes in organ architecture and cell viability were observed in the spleen and pLNs. JEV Ag staining was apparent in lymphoid organs, whereas limited levels of viral Ag was detected in the liver. Thus, it is conceivable that end-organ damage was secondary to ischemia and/or the toxic effects of cytokines rather than direct virus-induced apoptosis. Analogous to our study, IFN-I signal-incompetent mice in macrophages showed high lethality to *Listeria* infection by liver damage, which was coupled to high production of cytokines and chemokines [73]. Consistent with our assumption that pro-inflammatory cytokines contributed to the susceptibility of IFN-I signal-incompetent mice to JEV infection, administration of TNF-α and IL-6 blocking mAb in these mice prolonged survival following WNV infection but did not affect viral replication [74]. Furthermore, vasoactive TNF-α was suggested to cause hemorrhage-like syndrome via alternative pathway complement activation, possibly resulting in the production of C5a anaphylatoxin, because liver injury was minimized in C5-depleted IFN-I signal-incompetent mice following WNV infection [74]. Therefore, our speculation and these findings suggest that the excessive cytokine production of a “cytokine storm” in IFN-I signal-incompetent mice contributes to the injury of peripheral organs, but is not primarily responsible for the severe hemorrhage-like syndrome.

Flaviviruses including JEV, DenV, ZIKV, and WNV are closely related at the antigenic level. However, these flaviviruses cause different fatal diseases, typically encephalitis and vascular shock syndrome, depending on etiological agents [1]. For example, DenV infection causes DHF/dengue shock syndrome (DSS) similar to the hemorrhage-like disease seen in JEV infection in IFN-I signal-incompetent mice, whereas WNV and JEV cause acute encephalitis in IFN-I signal-competent mice. As with other flaviviruses, including DenV and WNV, JEV has been shown to inhibit IFN-I production and their signal from IFNAR1 by producing nonstructural (NS) proteins such as JEV NS5 and NS1 [34–36]. Among the NS proteins, the NS5 of flaviviruses is believed to play a crucial role in blocking IFN-I signaling. DenV and WNV NS5 potently block STAT2 activation and ZIKV NS5 is also known to inhibit both IFN-I production and its downstream signaling [60, 75, 76]. In the case of JEV infection, NS5 appears to reduce the phosphorylation of Tyk2 and STAT1 through a PTP-dependent mechanism [34, 35]. Viral encephalitis caused by WNV and JEV infection was well established in a murine model using IFN-I-competent mice, but the DHF/DSS model caused by DenV infection cannot yet simulate human vascular syndrome using IFN-I-competent mice [77]. To induce human-like DHF/DSS in a murine system, highly immunocompromised mice such as type I/II IFN (IFN-α/β/γ) receptor-deficient mice have been used in the mouse-adapted DenV strain [78]. This means that the different types of disease (e.g., encephalitis and vascular syndrome) may depend on the degree to which flaviviruses inhibit IFN-I signaling, even though the degree of IFN-I signaling inhibition by NS proteins derived from flaviviruses is unknown. Indeed, JEV infection is increasingly, but infrequently, reported as causing intracranial hemorrhage with JE [79, 80], and neurological signs of DenV infection are increasingly reported [81–83]. JEV is a proven neurotrophic virus and mainly targets neuronal cells in the CNS, whereas DenV is considered a non-neurotrophic virus. Approximately, one-half of all JE survivors have permanent neurological sequelae, but most patients even with neurological manifestations in DenV infection have an unexpected recovery with no obvious sequelae [81–83], which indicates that there are subtle differences in the progression of overlapping neurological signs caused by JEV and DenV. Therefore, the crucial role of IFN-I signaling in flavivirus infection should be deeply considered because two types of flaviviruses, JEV and DenV, cause overlapping yet subtly different disease patterns.

## Conclusions

Our data demonstrate that the intrinsic IFN-I signal in tissue-resident cells is critical in limiting the tissue tropism of viral dissemination following distal JEV inoculation, and the early and higher infection of circulating CD11b^+^Ly-6C^+^ monocytes at the inoculation site contributes to viral dissemination into the entire body at the early stage. Therefore, our study proposes a model of IFN-I signal-dependent cellular factors in viral tissue tropism to induce CNS neuroinflammation following distal JEV inoculation.

## Data Availability

The data supporting the conclusions of this article are included within the article. Original slides, photographs, and FACS dot-plots are retained. All reagents used in this study are available from scientific supply companies. The datasets used and analyzed during the current study are available from the corresponding author on reasonable request.

## Abbreviations

BM: Bone marrow
CBA: Cytokine bead array
CNS: Central nervous system
DCs: Dendritic cells
DenV: Dengue virus
DF: Dengue fever
DHF: Dengue hemorrhagic fever
dpi: Days post-infection
DSS: Dengue shock syndrome
IFN-I: Type I interferon
IFN-II: Type II interferon
ISGs: Interferon-stimulated genes
JEV: Japanese encephalitis virus
KO: Knock-out
PAMPs: Pathogen-associated molecular patterns
PRRs: Pathogen recognition receptors
TJs: Tight junctions
WNV: West Nile virus
ZIKV: Zika virus

## References

[1] Pierson TC, Diamond MS (2020). The continued threat of emerging flaviviruses. Nat Microbiol.

[2] Banerjee A, Tripathi A. Recent advances in understanding Japanese encephalitis. F1000Res. 2019;8. 10.12688/f1000research.19693.1 PMC6854871 31781366

[3] Heffelfinger JD, Li X, Batmunkh N, Grabovac V, Fiorditsa S, Liyanage JB, Pattamadilok S, Bahl S (2017). Japanese encephalitis surveillance and immunization – Asia and Western Pacific regions, 2016. MMWR Morb Mortal Wkly Rep.

[4] Center for disease. West Nile virus neuroinvasive disease cases reported to CDC by state of residence, 1999-2018. (assessed on Sep 20, 2020) http://www.cdc.gov/ncdod/dvbid/westnile/sure&control.

[5] Wilson MR (2013). Emerging viral infections. Curr Opin Neurol.

[6] Kimura T, Sasaki M, Okumura M, Kim E, Sawa H (2010). Flavivirus encephalitis: pathological aspects of mouse and other animal models. Vet Pathol.

[7] Choi JY, Kim JH, Hossain FMA, Uyangaa E, Park SO, Kim B, Kim K, Eo SK (2019). Indispensable role of CX3CR1(+) dendritic cells in regulation of virus-induced neuroinflammation through rapid development of antiviral immunity in peripheral lymphoid tissues. Front Immunol.

[8] Chen CJ, Ou YC, Lin SY, Raung SL, Liao SL, Lai CY, Chen SY, Chen JH (2010). Glial activation involvement in neuronal death by Japanese encephalitis virus infection. J Gen Virol.

[9] Ghoshal A, Das S, Ghosh S, Mishra MK, Sharma V, Koli P, Sen E, Basu A (2007). Proinflammatory mediators released by activated microglia induces neuronal death in Japanese encephalitis. Glia.

[10] Ghosh D, Basu A (2009). Japanese encephalitis-a pathological and clinical perspective. PLoS Negl Trop Dis.

[11] Wang ZY, Zhen ZD, Fan DY, Qin CF, Han DS, Zhou HN, et al. Axl deficiency promotes the neuroinvasion of Japanese encephalitis virus by enhancing IL-1alpha production from pyroptotic macrophages. J Virol. 2020;94(17). 10.1128/JVI.00602-20. 10.1128/JVI.00602-20 PMC7431807 32611752

[12] Larena M, Regner M, Lobigs M (2013). Cytolytic effector pathways and IFN-gamma help protect against Japanese encephalitis. Eur J Immunol.

[13] Kim JH, Patil AM, Choi JY, Kim SB, Uyangaa E, Hossain FM, Park SY, Lee JH, Eo SK (2016). CCR5 ameliorates Japanese encephalitis via dictating the equilibrium of regulatory CD4(+)Foxp3(+) T and IL-17(+)CD4(+) Th17 cells. J Neuroinflammation.

[14] Bardina SV, Lim JK (2012). The role of chemokines in the pathogenesis of neurotropic flaviviruses. Immunol Res.

[15] Saito T, Gale M (2007). Principles of intracellular viral recognition. Curr Opin Immunol.

[16] Ugolini M, Sander LE (2019). Dead or alive: how the immune system detects microbial viability. Curr Opin Immunol.

[17] Gack MU, Diamond MS (2016). Innate immune escape by dengue and West Nile viruses. Curr Opin Virol.

[18] Lazear HM, Diamond MS (2015). New insights into innate immune restriction of West Nile virus infection. Curr Opin Virol.

[19] Trinchieri G (2010). Type I interferon: friend or foe?. J Exp Med.

[20] Lazear HM, Pinto AK, Vogt MR, Gale M (2011). Diamond MS: beta interferon controls West Nile virus infection and pathogenesis in mice. J Virol.

[21] Daffis S, Suthar MS, Szretter KJ, Gale M, Diamond MS (2009). Induction of IFN-beta and the innate antiviral response in myeloid cells occurs through an IPS-1-dependent signal that does not require IRF-3 and IRF-7. PLoS Pathog.

[22] Lazear HM, Lancaster A, Wilkins C, Suthar MS, Huang A, Vick SC, Clepper L, Thackray L, Brassil MM, Virgin HW, Nikolich-Zugich J, Moses AV, Gale M, Früh K, Diamond MS (2013). IRF-3, IRF-5, and IRF-7 coordinately regulate the type I IFN response in myeloid dendritic cells downstream of MAVS signaling. PLoS Pathog.

[23] Tsai MH, Pai LM, Lee CK (2019). Fine-tuning of type I interferon response by STAT3. Front Immunol.

[24] Uehata T, Takeuchi O. RNA recognition and immunity-innate immune sensing and its posttranscriptional regulation mechanisms. Cells. 2020;9(7). 10.3390/cells9071701. 10.3390/cells9071701 PMC7407594 32708595

[25] Pinto AK, Daffis S, Brien JD, Gainey MD, Yokoyama WM, Sheehan KC, Murphy KM, Schreiber RD, Diamond MS (2011). A temporal role of type I interferon signaling in CD8+ T cell maturation during acute West Nile virus infection. PLoS Pathog.

[26] Szretter KJ, Brien JD, Thackray LB, Virgin HW, Cresswell P, Diamond MS (2011). The interferon-inducible gene viperin restricts West Nile virus pathogenesis. J Virol.

[27] Szretter KJ, Daniels BP, Cho H, Gainey MD, Yokoyama WM, Gale M, Virgin HW, Klein RS, Sen GC, Diamond MS (2012). 2’-O methylation of the viral mRNA cap by West Nile virus evades ifit1-dependent and -independent mechanisms of host restriction in vivo. PLoS Pathog.

[28] Lindqvist R, Mundt F, Gilthorpe JD, Wolfel S, Gekara NO, Kroger A, Overby AK (2016). Fast type I interferon response protects astrocytes from flavivirus infection and virus-induced cytopathic effects. J Neuroinflammation.

[29] Lucas TM, Richner JM, Diamond MS (2015). The interferon-stimulated gene Ifi27l2a restricts West Nile Virus infection and pathogenesis in a cell-type- and region-specific manner. J Virol.

[30] Han YW, Choi JY, Uyangaa E, Kim SB, Kim JH, Kim BS, Kim K, Eo SK (2014). Distinct dictation of Japanese encephalitis virus-induced neuroinflammation and lethality via triggering TLR3 and TLR4 signal pathways. PLoS Pathog.

[31] Kim SB, Choi JY, Kim JH, Uyangaa E, Patil AM, Park SY, Lee JH, Kim K, Han YW, Eo SK (2015). Amelioration of Japanese encephalitis by blockage of 4-1BB signaling is coupled to divergent enhancement of type I/II IFN responses and Ly-6C(hi) monocyte differentiation. J Neuroinflammation.

[32] Kim SB, Choi JY, Uyangaa E, Patil AM, Hossain FM, Hur J, Park SY, Lee JH, Kim K, Eo SK (2016). Blockage of indoleamine 2,3-dioxygenase regulates Japanese encephalitis via enhancement of type I/II IFN innate and adaptive T-cell responses. J Neuroinflammation.

[33] Lin RJ, Liao CL, Lin E, Lin YL (2004). Blocking of the alpha interferon-induced Jak-Stat signaling pathway by Japanese encephalitis virus infection. J Virol.

[34] Lin RJ, Chang BL, Yu HP, Liao CL, Lin YL (2006). Blocking of interferon-induced Jak-Stat signaling by Japanese encephalitis virus NS5 through a protein tyrosine phosphatase-mediated mechanism. J Virol.

[35] Ye J, Chen Z, Li Y, Zhao Z, He W, Zohaib A, et al. Japanese encephalitis virus NS5 inhibits type I interferon (IFN) production by blocking the nuclear translocation of IFN regulatory factor 3 and NF-kappaB. J Virol. 2017;91(8). 10.1128/JVI.00039-17. 10.1128/JVI.00039-17 PMC5375679 28179530

[36] Zhou D, Li Q, Jia F, Zhang L, Wan S, Li Y, Song Y, Chen H, Cao S, Ye J (2020). The Japanese encephalitis virus NS1’ protein inhibits type I IFN production by targeting MAVS. J Immunol.

[37] Cho H, Proll SC, Szretter KJ, Katze MG, Gale M, Diamond MS (2013). Differential innate immune response programs in neuronal subtypes determine susceptibility to infection in the brain by positive-stranded RNA viruses. Nat Med.

[38] Schoggins JW, Wilson SJ, Panis M, Murphy MY, Jones CT, Bieniasz P, Rice CM (2011). A diverse range of gene products are effectors of the type I interferon antiviral response. Nature.

[39] Uyangaa E, Kim JH, Patil AM, Choi JY, Kim SB, Eo SK (2015). Distinct upstream role of type I IFN signaling in hematopoietic stem cell-derived and epithelial resident cells for concerted recruitment of Ly-6Chi monocytes and NK cells via CCL2-CCL3 cascade. PLoS Pathog.

[40] George JA, Park SO, Choi JY, Uyangaa E, Eo SK (2020). Double-faced implication of CD4(+) Foxp3(+) regulatory T cells expanded by acute dengue infection via TLR2/MyD88 pathway. Eur J Immunol.

[41] Macartney KK, Baumgart DC, Carding SR, Brubaker JO, Offit PA (2000). Primary murine small intestinal epithelial cells, maintained in long-term culture, are susceptible to rotavirus infection. J Virol.

[42] Liu S, Stolz DB, Sappington PL, Macias CA, Killeen ME, Tenhunen JJ, Delude RL, Fink MP (2006). HMGB1 is secreted by immunostimulated enterocytes and contributes to cytomix-induced hyperpermeability of Caco-2 monolayers. Am J Phys Cell Phys.

[43] Li WC, Ralphs KL, Tosh D (2010). Isolation and culture of adult mouse hepatocytes. Methods Mol Biol.

[44] Bridges BC, Hardison D, Pietsch J (2013). A case series of the successful use of ECMO, continuous renal replacement therapy, and plasma exchange for thrombocytopenia-associated multiple organ failure. J Pediatr Surg.

[45] Latimer G, Corriveau C, DeBiasi RL, Jantausch B, Delaney M, Jacquot C, Bell M, Dean T (2020). Cardiac dysfunction and thrombocytopenia-associated multiple organ failure inflammation phenotype in a severe paediatric case of COVID-19. Lancet Child Adolesc Health.

[46] Arem R (1989). Hypoglycemia associated with renal failure. Endocrinol Metab Clin N Am.

[47] Naylor JM, Kronfeld DS (1985). In vivo studies of hypoglycemia and lactic acidosis in endotoxic shock. Am J Phys.

[48] Hargrove DM, Bagby GJ, Lang CH, Spitzer JJ (1988). Adrenergic blockade dose not abolish elevated glucose turnover during bacterial infection. Am J Phys.

[49] Maitra SR, Gestring ML, El-Maghrabi MR, Lang CH, Henry MC (1999). Endotoxin-induced alterations in hepatic glucose-6-phospahatase activity and gene expression. Mol Cell Biochem.

[50] Srikiatkhachorn A, Spiropoulou CF (2014). Vascular events in viral hemorrhagic fevers: a comparative study of dengue and hantaviruses. Cell Tissue Res.

[51] Chao CH, Wu WC, Lai YC, Tsai PJ, Perng GC, Lin YS, Yeh TM (2019). Dengue virus nonstructural protein 1 activates platelets via Toll-like receptor 4, leading to thrombocytopenia and hemorrhage. PLoS Pathog.

[52] de Mast Q, de Groot PG (2019). Serotonin, key to thrombocytopenia in dengue?. Blood.

[53] Chanthick C, Suttitheptumrong A, Rawarak N, Pattanakitsakul SN. Transcytosis involvement in transport system and endothelial permeability of vascular leakage during dengue virus infection. Viruses. 2018;10(2). 10.3390/v10020069. 10.3390/v10020069 PMC5850376 29419739

[54] Phanthanawiboon S, Limkittikul K, Sakai Y, Takakura N, Saijo M, Kurosu T (2016). Acute systemic infection with dengue virus leads to vascular leakage and death through tumor necrosis factor-alpha and Tie2/angiopoietin signaling in mice lacking type I and II interferon receptors. PLoS One.

[55] Daly C, Rollins BJ (2003). Monocyte chemoattractant protein-1 (CCL2) in inflammatory disease and adaptive immunity: therapeutic opportunities and controversies. Microcirculation.

[56] Satpathy AT, Wu X, Albring JC, Murphy KM (2012). Re(de)fining the dendritic cell lineage. Nat Immunol.

[57] Italiani P, Boraschi D (2014). From monocytes to M1/M2 macrophages: phenotypical vs. functional differentiation. Front Immunol.

[58] Ransohoff RM, Brown MA (2012). Innate immunity in the central nervous system. J Clin Invest.

[59] Terry RL, Getts DR, Deffrasnes C, van Vreden C, Campbell IL, King NJ (2012). Inflammatory monocytes and the pathogenesis of viral encephalitis. J Neuroinflammation.

[60] Grant A, Ponia SS, Tripathi S, Balasubramaniam V, Miorin L, Sourisseau M, Schwarz MC, Sanchez-Seco MP, Evans MJ, Best SM, Garcia-Sastre A (2016). Zika virus targets human STAT2 to inhibit type I interferon signaling. Cell Host Microbe.

[61] Lux D, Alakbarzade V, Bridge L, Clark CN, Clarke B, Zhang L, Khan U, Pereira AC (2020). The association of neutrophil-lymphocyte ratio and lymphocyte-monocyte ratio with 3-month clinical outcome after mechanical thrombectomy following stroke. J Neuroinflammation.

[62] Kolattukudy PE, Niu J (2012). Inflammation, endoplasmic reticulum stress, autophagy, and the monocyte chemoattractant protein-1/CCR2 pathway. Circ Res.

[63] Iijima N, Mattei LM, Iwasaki A (2011). Recruited inflammatory monocytes stimulate antiviral Th1 immunity in infected tissue. Proc Natl Acad Sci U S A.

[64] Uyangaa E, Choi JY, Patil AM, Hossain FMA, Park SO, Kim B, Kim K, Eo SK (2018). Dual TLR2/9 recognition of herpes simplex virus infection is required for recruitment and activation of monocytes and NK cells and restriction of viral dissemination to the central nervous system. Front Immunol.

[65] Tisoncik JR, Korth MJ, Simmons CP, Farrar J, Martin TR, Katze MG (2012). Into the eye of the cytokine storm. Microbiol Mol Biol Rev.

[66] Wang H, Ma S (2008). The cytokine storm and factors determining the sequence and severity of organ dysfunction in multiple organ dysfunction syndrome. Am J Emerg Med.

[67] Wang Q, Fang P, He R, Li M, Yu H, Zhou L, Yi Y, Wang F, Rong Y, Zhang Y (2020). O-GlcNAc transferase promotes influenza A virus-induced cytokine storm by targeting interferon regulatory factor-5. Sci Adv.

[68] Hossain FMA, Choi JY, Uyangaa E, Park SO, Eo SK (2019). The interplay between host immunity and respiratory viral infection in asthma exacerbation. Immune Netw.

[69] Roshanravan N, Seif F, Ostadrahimi A, Pouraghaei M, Ghaffari S (2020). Targeting cytokine storm to manage patients with COVID-19: a mini-review. Arch Med Res.

[70] Lee P, Kim DJ (2020). Newly emerging human coronaviruses: animal models and vaccine research for SARS, MERS, and COVID-19. Immune Netw.

[71] Tan GK, Ng JK, Trasti SL, Schul W, Yip G, Alonso S (2010). A non mouse-adapted dengue virus strain as a new model of severe dengue infection in AG129 mice. PLoS Negl Trop Dis.

[72] Srikiatkhachorn A, Mathew A, Rothman AL (2017). Immune-mediated cytokine storm and its role in severe dengue. Semin Immunopathol.

[73] Kernbauer E, Maier V, Stoiber D, Strobl B, Schneckenleithner C, Sexl V, Reichart U, Reizis B, Kalinke U, Jamieson A, Müller M, Decker T (2012). Conditional Stat1 ablation reveals the importance of interferon signaling for immunity to Listeria monocytogenes infection. PLoS Pathog.

[74] Pinto AK, Ramos HJ, Wu X, Aggarwal S, Shrestha B, Gorman M, Kim KY, Suthar MS, Atkinson JP, Gale M, Diamond MS (2014). Deficient IFN signaling by myeloid cells leads to MAVS-dependent virus-induced sepsis. PLoS Pathog.

[75] Laurent-Rolle M, Boer EF, Lubick KJ, Wolfinbarger JB, Carmody AB, Rockx B, Liu W, Ashour J, Shupert WL, Holbrook MR, Barrett AD, Mason PW, Bloom ME, García-Sastre A, Khromykh AA, Best SM (2010). The NS5 protein of the virulent West Nile virus NY99 strain is a potent antagonist of type I interferon-mediated JAK-STAT signaling. J Virol.

[76] Best SM. The many faces of the flavivirus NS5 protein in antagonism of type I interferon signaling. J Virol. 2017;91(3). 10.1128/JVI.01970-16. 10.1128/JVI.01970-16 PMC5244349 27881649

[77] Hassert M, Brien JD, Pinto AK (2019). Mouse models of heterologous flavivirus immunity: a role for cross-reactive T cells. Front Immunol.

[78] Watanabe S, Vasudevan SG (2014). Evaluation of dengue antiviral candidates in vivo in mouse model. Methods Mol Biol.

[79] Ma'roef CN, Dhenni R, Megawati D, Fadhilah A, Lucanus A, Artika IM, Masyeni S, Lestarini A, Sari K, Suryana K (2020). Japanese encephalitis virus infection in non-encephalitic acute febrile illness patients. PLoS Negl Trop Dis.

[80] Feng Q, Chen Q, Bi X, Yu S, Wang J, Sun X, Ren C, Liu H, Guan L (2019). Severe Japanese encephalitis with multiple intracranial hemorrhages: a case report. Medicine.

[81] Kutiyal AS, Malik C, Hyanki G (2017). Dengue haemorrhagic encephalitis: rare case report with review of literature. J Clin Diagn Res.

[82] Weerasinghe WS, Medagama A (2019). Dengue hemorrhagic fever presenting as encephalitis: a case report. J Med Case Rep.

[83] Sarkar R, Paul R, Thakur I, Ghosh R, Singh S, Mani A, Sau TJ, Lahiri G (2019). Encephalitis due to dengue virus infection mimicking Japanese B encephalitis: two case reports. J Assoc Physicians India.

